# Life's Essential 8 and carotid artery plaques: the Swedish cardiopulmonary bioimage study

**DOI:** 10.3389/fcvm.2023.1173550

**Published:** 2023-06-22

**Authors:** Ángel Herraiz-Adillo, Viktor H. Ahlqvist, Sara Higueras-Fresnillo, Daniel Berglind, Patrik Wennberg, Cecilia Lenander, Bledar Daka, Mattias Ekstedt, Johan Sundström, Francisco B. Ortega, Carl Johan Östgren, Karin Rådholm, Pontus Henriksson

**Affiliations:** ^1^ Department of Health, Medicine and Caring Sciences, Linköping University, Linköping, Sweden; ^2^ Department of Global Public Health, Karolinska Institutet, Stockholm, Sweden; ^3^ Department of Preventive Medicine and Public Health, Universidad Autónoma de Madrid, Madrid, Spain; ^4^ Centre for Epidemiology and Community Medicine, Region Stockholm, Stockholm, Sweden; ^5^ Department of Public Health and Clinical Medicine, Family Medicine, Umeå University, Umeå, Sweden; ^6^ Department of Clinical Sciences in Malmö, Centre for Primary Health Care Research, Lund University, Lund, Sweden; ^7^ School of Public Health and Community Medicine, Institute of Medicine, Sahlgrenska Academy, University of Gothenburg, Gothenburg, Sweden; ^8^ Centre of Medical Image Science and Visualization (CMIV), Linköping University, Linköping, Sweden; ^9^ Clinical Epidemiology Unit, Department of Medical Sciences, Uppsala University, Uppsala, Sweden; ^10^ The George Institute for Global Health, University of New South Wales, Sydney, NSW, Australia; ^11^ Department of Physical Education and Sports, Faculty of Sport Sciences, Sport and Health, University Research Institute (iMUDS), University of Granada; CIBERobn Physiopathology of Obesity and Nutrition, Granada, Spain; ^12^ Faculty of Sport and Health Sciences, University of Jyväskylä, Jyväskylä, Finland

**Keywords:** carotid atherosclerosis, Doppler ultrasound, health promotion, healthy lifestyle, population-based, SCAPIS project

## Abstract

**Background:**

To quantify cardiovascular health (CVH), the American Heart Association (AHA) recently launched an updated construct of the “Life's Simple 7” (LS7) score, the “Life's Essential 8” (LE8) score. This study aims to analyse the association between both CVH scores and carotid artery plaques and to compare the predictive capacity of such scores for carotid plaques.

**Methods:**

Randomly recruited participants aged 50–64 years from the Swedish CArdioPulmonary bioImage Study (SCAPIS) were analysed. According to the AHA definitions, two CVH scores were calculated: i) the LE8 score (0, worst CVH; 100, best CVH) and two different versions of the LS7 score [(0–7) and (0–14), 0 indicating the worst CVH]. Ultrasound-diagnosed carotid plaques were classified as no plaque, unilateral, and bilateral plaques. Associations were studied by adjusted multinomial logistic regression models and adjusted (marginal) prevalences, while comparison between LE8 and LS7 scores was performed through receiver operating characteristic (ROC) curves.

**Results:**

After exclusions, 28,870 participants remained for analysis (50.3% women). The odds for bilateral carotid plaques were almost five times higher in the lowest LE8 (<50 points) group [OR: 4.93, (95% CI: 4.19–5.79); adjusted prevalence 40.5%, (95% CI: 37.9–43.2)] compared to the highest LE8 (≥80 points) group [adjusted prevalence 17.2%, (95% CI: 16.2–18.1)]. Also, the odds for unilateral carotid plaques were more than two times higher in the lowest LE8 group [OR: 2.14, (95% CI: 1.82–2.51); adjusted prevalence 31.5%, (95% CI: 28.9–34.2)] compared to the highest LE8 group [adjusted prevalence 29.4%, (95% CI: 28.3–30.5)]. The areas under ROC curves were similar between LE8 and LS7 (0–14) scores: for bilateral carotid plaques, 0.622 (95% CI: 0.614–0.630) vs. 0.621 (95% CI: 0.613–0.628), *P* = 0.578, respectively; and for any carotid plaque, 0.602 (95% CI: 0.596–0.609) vs. 0.600 (95% CI: 0.593–0.607), *P* = 0.194, respectively.

**Conclusion:**

The new LE8 score showed inverse and dose-response associations with carotid plaques, particularly bilateral plaques. The LE8 did not outperform the conventional LS7 score, which showed similar ability to predict carotid plaques, especially when scored as 0–14 points. We conclude that both the LE8 and LS7 may be useful in clinical practice for monitoring CVH status in the adult population.

## Introduction

Cardiovascular disease, including coronary heart disease and cerebrovascular disease, continues to be the leading cause of morbidity and mortality globally (1). The American Heart Association (AHA) estimated that 7.08 million deaths worldwide were attributable to cerebrovascular disease in 2020, and the expected prevalence of people living with stroke is on the rise due to population aging and improved survival rate (2, 3). Overall, stroke accounts for one out of every six cardiovascular deaths (1), and it is a leading cause of disability (2, 4), making it a major public health problem.

Atherosclerosis is firmly established as a leading cause of clinical cerebrovascular events (1), and carotid plaques are feasible and useful markers of cardiovascular risk since they are indicators of the general atherosclerotic burden on the arterial tree (5). Thus, the burden and characteristics of atherosclerosis in the carotid arteries are associated with the atherosclerosis in other regions, such as the coronary arteries (6), and the grade of carotid stenosis has proven to be an independent risk factor for cardiovascular and all-cause mortality (5). Additionally, carotid plaques play an important role in the development of ischemic stroke, not only as a proxy of the atherosclerotic burden, but also due to formation and release of thromboemboli following carotid plaque rupture (7).

Consequently, to combat the cardiovascular burden, public health policies based on primordial prevention have been advocated. In this sense, the AHA defined the construct “ideal cardiovascular health” or “Life's Simple 7” (LS7) in 2010, which meant a crucial shift from cardiovascular disease management to positive cardiovascular health (CVH) promotion (8). During the last decade, LS7 proved to be of great utility in monitoring individual and public CVH and confirmed a strong inverse dose-response association between the number of components at the ideal level and cardiovascular events, cardiovascular mortality, all-cause mortality, and different non-cardiovascular outcomes (9–12). However, LS7 has some limitations, specifically a lack of sensitivity to detect interindividual differences and intraindividual change over time in CVH assessment. To overcome these limitations, in June 2022, the AHA defined a new construct called “Life's Essential 8” (LE8) (13). Compared to the older LS7, the newer LE8 also includes sleep health and updated definitions or scores for the previous 7 components: diet, physical activity, nicotine exposure, body mass index (BMI), blood lipids, blood glucose, and blood pressure.

In the last decade, LS7 has accumulated evidence of its association with functional and structural indicators of carotid atherosclerosis both in cross-sectional and prospective studies (14–16), especially in Chinese populations. Arguably, LE8 should outperform LS7 as a monitoring tool for carotid atherosclerosis, since the adoption of LE8 was intended to improve cardiovascular monitoring (13) and includes sleep health, a proven cardiovascular risk factor (17). However, to our knowledge, this remains to be formally tested; there have been no previous studies that have analysed the association between the recently developed LE8 score and ultrasound-diagnosed carotid plaques, despite the potential of this new AHA's CVH construct to inform about subclinical atherosclerosis. Therefore, the aims of this study were to investigate the association of the LE8 and LS7 scores with ultrasound-diagnosed carotid plaques in a middle-aged general population and to compare the predictive capacity of such scores for carotid plaques.

## Materials and methods

### SCAPIS

Detailed design and methods of the Swedish CArdioPulmonary bioImage Study (SCAPIS) have been published elsewhere (18). In brief, SCAPIS is a collaborative project involving different universities and university hospitals in Sweden (Gothenburg, Linköping, Malmö/Lund, Stockholm, Umeå, and Uppsala) aiming to predict and prevent cardiovascular and pulmonary disease. This population-based study randomly recruited 30,154 aged 50–64 years participants (overall participation rate = 50.3%), which were characterized by detailed imaging and functional analyses including thoracoabdominal computed tomography and carotid ultrasound.

### Study population

**Figure 1:**
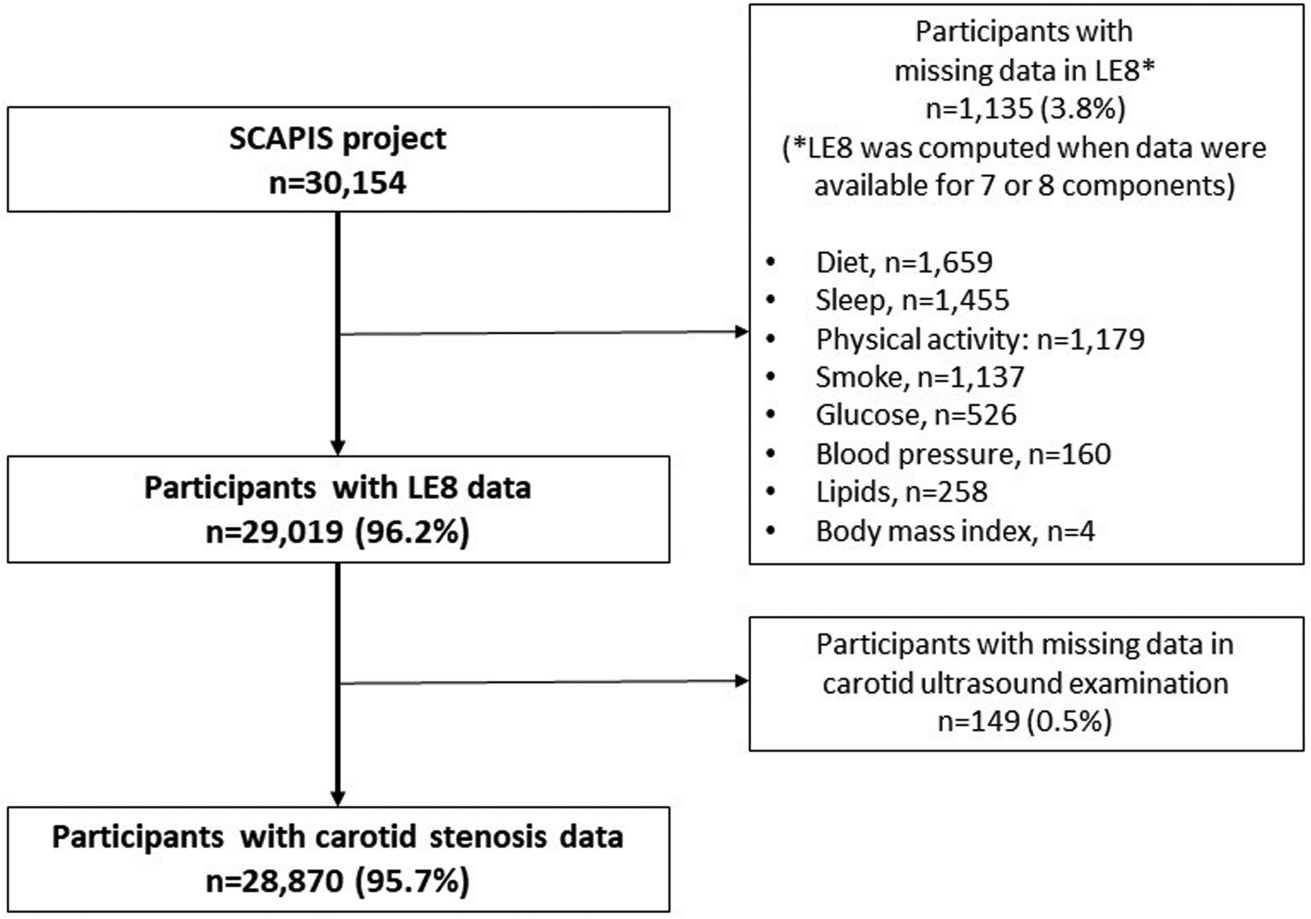
Flow chart of the study.

This study included participants from SCAPIS with necessary data to calculate LE8 and LS7 scores, and with available ultrasound images in both left and right carotid arteries. Figure 1 depicts a flow chart for the study. In brief, of the 30,154 participants included in the SCAPIS project, 1,135 (3.8%) participants had missing data on the LE8 score and, in addition, 149 (0.5%) participants did not undergo ultrasound carotid examination, leaving a final sample size of 28,870 (95.7%) participants. The Swedish Ethical Review Authority granted ethical approval for this work (reference numbers: 2021-06408-01 and 2022-04375-02). All participants provided written informed consent.

### Cardiovascular health scores

#### Life's Essential 8

We constructed a LE8 score to include the 8 components as defined by the AHA (13). Supplementary Appendix S1 details the measurement and calculation of health factors [BMI, non-high density lipoprotein cholesterol (non-HDL), blood glucose and blood pressure] and health behaviours (diet, physical activity, nicotine exposure, and sleep health) in the LE8 score.

Briefly, LE8 factors were measured using standardized clinical and laboratory techniques. Regarding LE8 behaviors, questionnaires were used to measure nicotine exposure, sleep health (considering hours of sleep per night, breathing problems during sleep, sleep apnea and sleep apnea treatment) and diet [web-based MiniMeal-Q to calculate adherence to the Mediterranean Eating Pattern for Americans (19)]. Physical activity was measured through tri-axial accelerometry (Actigraph GT3X+, wGT3X+, and wGT3X-BT) with cut-offs to classify moderate and vigorous physical activity set at ≥2,690–6,166 and ≥6,167 counts per minute, respectively (20).

In accordance with the AHA recommendations, a total score from 0 to 100 (ideal CVH) was calculated based on 7 or 8 components within LE8 (i.e., an individual needed to have at least 7 components recorded) through the unweighted average of all present components. To aid clinical interpretation, the total LE8 score was categorized in 5 groups as follows: <50, 50–59.9, 60–69.9, 70–79.9, and 80–100 points. This categorization is comparable to that proposed by the AHA (80–100, high CVH; 50–79, moderate CVH; and 0–49 points, low CVH) (13), but we also decided to split the moderate CVH group into 3 categories to comprehensively depict the association between LE8 and carotid plaques.

Similarly, two separate scores ranging from 0 to 100 were calculated and categorized for LE8 factors and LE8 behaviors.

#### Life's Simple 7

Measurements used in LS7 score were similar to those used in LE8 score (apart from sleep health that was not included in LS7). Supplementary Appendix S1 details the measurement and calculation of health factors (total cholesterol, blood glucose, and blood pressure) and health behaviors (BMI, diet, physical activity, and smoking status) in LS7 score. It fulfils the AHA recommendations (8) and has been published elsewhere (21). The diet component was consistent with the Dietary Approaches to Stop Hypertension (DASH) eating plan (22).

Two different LS7 scores were derived: the LS7 (0–7) score, which is the sum of the number of LS7 components at ideal level, producing a total 0–7 score (0, the lowest CVH; 7, the highest CVH); and the LS7 (0–14) score, which is the sum of the scores of LS7 components when rated as: 0 (poor CVH), 1 (intermediate CVH), and 2 (ideal CVH), generating a total 0–14 score (0, the lowest CVH; 14, the highest CVH).

### Ultrasound carotid artery plaques

The detailed imaging protocol for SCAPIS, which meets relevant guidelines, has been published elsewhere (18). All the images regarding carotid arteries were recorded using a standardized protocol with a Siemens Acuson S2000 ultrasound scanner equipped with a 9L4 linear transducer (Siemens, Forchheim, Germany) and analysed by regularly trained operators. The presence of extracranial carotid plaques was analysed through two-dimensional greyscale ultrasound images in the common carotid artery, bulb, and in the internal carotid artery for both right and left carotid arteries. Subjects with valid readings in both right and left carotid arteries were included in the analysis.

In consonance with the Mannheim consensus, carotid plaque was defined as “any focal structure that encroaches into the arterial lumen of at least 0.5 mm or 50% of the surrounding intima media thickness value or demonstrates a thickness >1.5 mm as measured from the media-adventitia interface to the intima-lumen interface” (23). Participants regarding carotid plaques were classified as having either no plaque, unilateral plaque/s, or bilateral plaques.

### Statistical analysis

Means and standard deviations were presented for continuous variables, and frequencies and percentages were presented for categorical variables. Multinomial logistic regression models were used to analyse the associations between LE8 and LS7 scores and carotid plaques: Model 1, unadjusted; Model 2, adjusted for age, sex, and study site (Gothenburg, Linköping, Malmö/Lund, Stockholm, Umeå, and Uppsala); and Model 3, adjusted for age, sex, study site, alcohol intake (frequency, and number of drinks in a typical day), educational status (attained highest level of education), current marital status, and presence of cardiovascular disease (self-reported myocardial infarction, coronary artery bypass grafting, percutaneous coronary intervention, stroke, or peripheral arterial disease). Model 2 was chosen as the main analysis, since it includes only basic demographic details, and may therefore be easily replicated.

To improve clinical interpretation, we estimated the adjusted (marginal) prevalence of carotid plaques indicators across CVH scores, which will be henceforth referred as “adjusted prevalence”. The capacity to predict carotid plaques for LE8 and LS7 [using both the LS7 (0–7) and the LS7 (0–14) scores] was analysed by comparing unadjusted receiver operating characteristic (ROC) curves through DeLong tests. Reclassification and discrimination were examined with the categorical Net Reclassification Index (NRI) and Integrated Discrimination Improvement (IDI) considering covariates in Model 2. For NRI, we set the threshold level for the risk categories at 50%.

The relationship between LE8, LS7 (0–7) and LS7 (0–14) was also modelled by restricted cubic splines of binary logistic models adjusted by age, sex and site.

In the primary analysis, we performed a complete case analysis on all included 28,870 participants. In addition, we performed an extreme scenario sensitivity analysis to corroborate the robustness of our findings. As such, we considered participants with missing data on either LE8 score or carotid plaque as having the best or the worst possible result, i.e., 0 (worst) or 100 (best) for LS8 score, and “no plaque” (best) or “bilateral plaques” (worst) for carotid plaques. In addition, we performed a secondary sensitivity analysis considering: (1) exclusion of participants with previous stroke, (2) exclusion of participants with previous cardiovascular diseases (self-reported myocardial infarction, coronary artery bypass grafting, percutaneous coronary intervention, stroke, or peripheral arterial disease intervention), and (3) inclusion of participants with data on all 8 components of the LE8 (instead of at least 7, as in the primary analysis).

All statistical tests were two-sided and *P* < 0.05 was considered statistically significant. Analyses were conducted using IBM-SPSS-28 (Armonk, NY: IBMCorp.) and Stata 17 (StataCorp. 2021).

## Results

### Descriptive statistics

Of a total sample of 30,154 participants, 4.3% were excluded due to a lack of data, leaving 28,870 participants for the analysis (Figure 1). In general, those participants excluded from the study exhibited slightly higher levels of cardiovascular risk factors and carotid atherosclerosis, and lower levels of CVH scores (Supplementary Table S1). Supplementary Appendix S2 contains Supplementary Tables and Figures.

Table 1 depicts the characteristics of the study population stratified by sex. The mean age of participants was 57.5 years, and 51.5% were females. The mean for LE8, LS7 (0–7) and LS7 (0–14) scores were 70.6, 3.3 and 9.1 points, respectively. Regarding LE8, 4.2% scored <50 points, 13.6% scored 50–59.9, 27.6% scored 60–69.9, 31.1% scored 70–79.9 and 23.5% scored ≥80 points. Regarding atherosclerosis, unilateral and bilateral carotid plaques were present in 29.7% and 25.3% of participants, respectively. Overall, women had higher levels of CVH and less carotid plaques than men (no carotid plaque, 51.0% vs. 38.5% in women and men, respectively).

**Table 1:** Clinical characteristics of the study sample by sex.

	Total *n* = 28,870	Women *n* = 14,862 (51.5%)	Men *n* = 14,008 (48.5%)
Age and cardiovascular risk factors			
Age, y	57.5 (4.3)	57.5 (4.3)	57.5 (4.4)
BMI, kg/m^2^	26.9 (4.4)	26.5 (4.8)	27.4 (3.9)
Obesity, *n* (%)	6,126 (21.2)	3,036 (20.4)	3,090 (22.1)
Total cholesterol, mg/dl	212.2 (40.7)	218.2 (39.5)	205.8 (40.9)
HDL cholesterol, mg/dl	63.1 (19.3)	71.0 (19.2)	54.6 (15.3)
LDL cholesterol, mg/dl	133.0 (37.3)	133.3 (37.2)	132.6 (37.5)
Hypercholesterolemia, *n* (%)^a^	3,339 (11.7)	1,392 (9.5)	1,947 (14.2)
Systolic blood pressure, mmHg	125.8 (17.0)	123.1 (17.8)	128.7 (15.6)
Diastolic blood pressure, mmHg	77.5 (10.5)	76.6 (10.8)	78.5 (10.1)
Hypertension, *n* (%)^a^	6,461 (22.7)	3,079 (21.0)	3,382 (24.6)
Fasting glucose, mg/dl	103.2 (19.8)	99.9 (17.1)	106.7 (21.8)
HbA1c, mmol/mol	36.5 (6.4)	36.2 (5.6)	36.8 (7.1)
Diabetes mellitus, *n* (%)^a^	1,245 (4.4)	459 (3.1)	786 (5.7)
Moderate-vigorous physical activity, min/day	55.9 (29.8)	54.0 (28.0)	57.9 (31.4)
LE8 diet (0–100) score	41.1 (16.1)	44.6 (16.2)	37.3 (15.0)
Smoking, *n* (%)			
Current	3,665 (12.8)	1,883 (12.8)	1,782 (12.8)
Ex-smoker	10,393 (36.4)	5,715 (38.9)	4,678 (33.7)
Never	14,516 (50.8)	7,098 (48.3)	7,418 (53.5)
Alcohol intake, frequency			
Never	2,552 (8.9)	1,522 (10.3)	1,030 (7.4)
Monthly or less	4,421 (15.5)	2,630 (17.8)	1,791 (12.9)
2–4 times a month	10,951 (38.3)	5,625 (38.2)	5,326 (38.4)
2–3 times a week	8,606 (30.1)	4,213 (28.6)	4,393 (31.7)
≥4 times a week	2,070 (7.2)	744 (5.0)	1,326 (9.6)
Social factors			
Education level			
Unfinished primary school	175 (0.6)	86 (0.6)	89 (0.6)
Primary school	2,455 (8.6)	1,097 (7.4)	1,358 (9.8)
Secondary school	13,069 (45.6)	6,311 (42.7)	6,758 (48.6)
University degree	12,984 (45.3)	7,285 (49.3)	5,699 (41.0)
Current marital status			
Single	3,832 (13.4)	2,039 (13.8)	1,793 (12.9)
Divorced	3,178 (11.1)	2,047 (13.9)	1,131 (8.2)
Married	21,137 (73.9)	10,300 (69.9)	10,837 (78.1)
Widow	473 (1.7)	357 (2.4)	116 (0.8)
Cardiovascular health scores			
Life's Essential 8 score			
<50	1,207 (4.2)	495 (3.3)	712 (5.1)
50–59.9	3,920 (13.6)	1,647 (11.1)	2,273 (16.2)
60–69.9	7,975 (27.6)	3,517 (23.7)	4,458 (31.8)
70–79.9	8,975 (31.1)	4,686 (31.5)	4,289 (30.6)
≥80	6,793 (23.5)	4,517 (30.4)	2,276 (16.2)
LE8 (0–100) score	70.6 (11.6)	72.6 (11.7)	68.5 (11.1)
Life's Simple 7 score			
LS7 (0–7) score	3.3 (1.3)	3.5 (1.3)	3.0 (1.2)
LS7 (0–14) score	9.1 (2.0)	9.5 (2.0)	8.8 (1.9)
Carotid plaques			
No plaque	12,974 (44.9)	7,576 (51.0)	5,398 (38.5)
Unilateral	8,583 (29.7)	4,296 (28.9)	4,287 (30.6)
Bilateral	7,313 (25.3)	2,990 (20.1)	4,323 (30.9)

BMI, body mass index; HDL, high density lipoprotein; LDL, low density lipoprotein; LE8, Life's Essential 8 score; LS7, Life's Simple 7 score.

Data refer to mean (standard deviation) and frequencies (percentage).

^a^ Sample size in self-reposted hypertension, hypercholesterolemia and diabetes mellitus, *n *= 28,419.

#### Life's Essential 8 and carotid plaques

**Figure 2:**
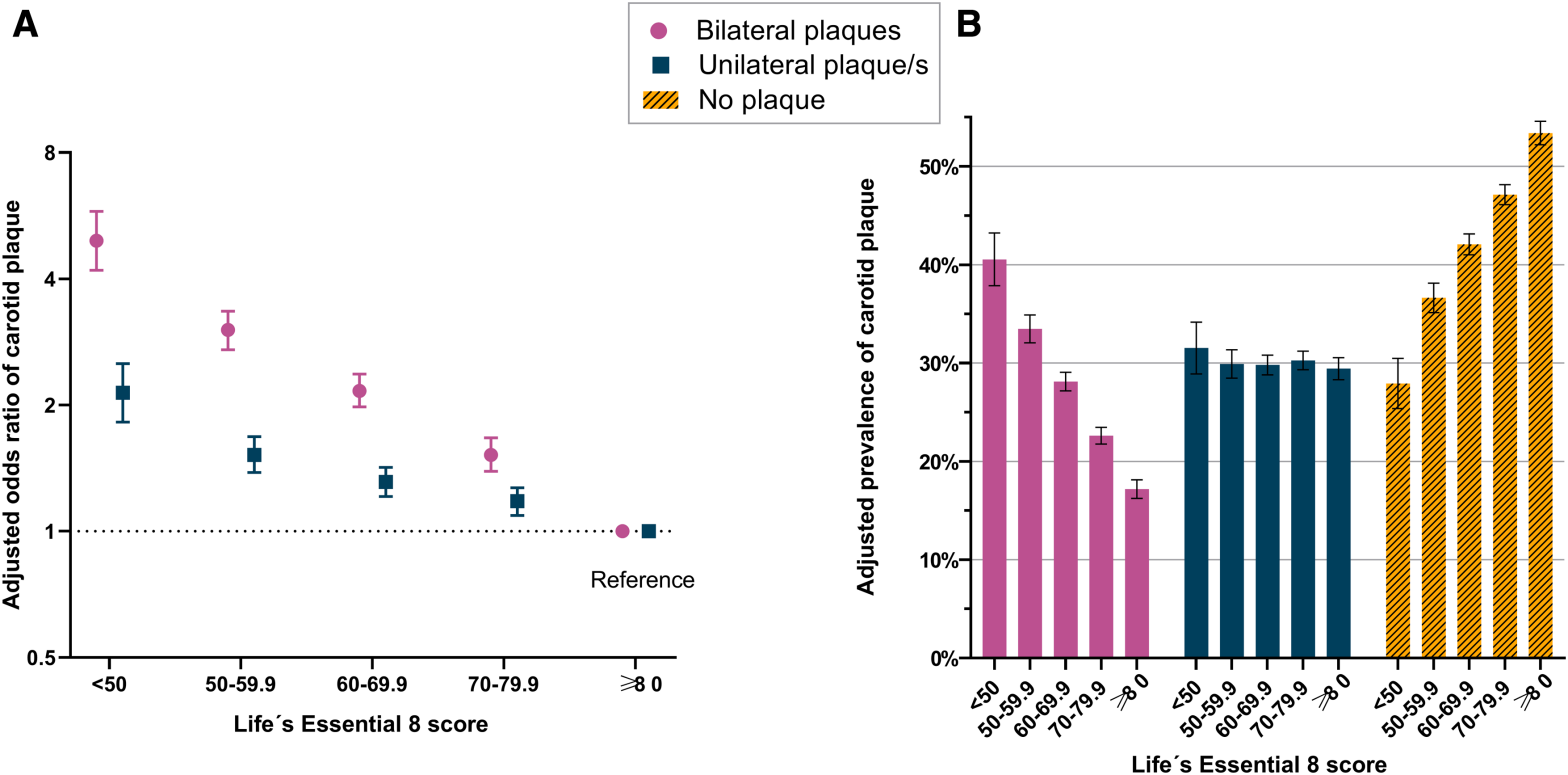
Life's Essential 8 and Carotid plaques. Panel A illustrates the multinomial regression model to estimate odds ratios of unilateral carotid plaque and bilateral carotid plaques across Life's Essential 8 scores (adjusted for sex, age, and study site). Panel B illustrates the adjusted prevalences of unilateral carotid plaque/s and bilateral carotid plaques across Life's Essential 8 scores (adjusted for sex, age, and study site).

Overall, having low LE8 scores was strongly and inversely associated with the presence of carotid plaques, particularly bilateral plaques (Figure 2 and Supplementary Tables S2,S3). Thus, the odds for bilateral plaques was almost five times higher in the lowest LE8 (<50 points) group [OR: 4.93, (95% CI: 4.19–5.79); adjusted prevalence 40.5%, (95% CI: 37.9–43.2)] compared to the highest LE8 (≥80 points) group [adjusted prevalence 17.2%, (95% CI: 16.2–18.1)]. Similarly, the odds for unilateral plaques was more than two times higher in the lowest LE8 group [OR: 2.14, (95% CI: 1.82–2.51); adjusted prevalence 31.5%, (95% CI: 28.9–34.2)] compared to the highest LE8 group [adjusted prevalence 29.4%, (95% CI: 28.3–30.5)]. Supplementary Figure S1 depicts the association between LE8 and carotid plaques through restricted cubic splines. Supplementary Table S4 depicts the association between continuous Life's Essential 8 (0–100 points) and carotid plaques.

Both health factors and health behaviors within the LE8 concept were strongly associated with carotid plaques, and the associations were particularly pronounced for health factors and bilateral carotid plaques (all *P* < 0.05), (Supplementary Table S5). In a separate analysis considering the individual 8 components in LE8, all components but sleep and diet were associated with carotid plaques (Supplementary Table S6).

#### Life's Simple 7 and carotid plaques

As in LS8, there were strong and inverse associations between LS7 (0–7) scores and carotid plaques, particularly for bilateral plaques (Supplementary Figure S2 and Supplementary Tables S7,S8). The odds of having bilateral carotid plaques was more than four times higher in the lowest LS7 (≤1 point) group [OR: 4.30, (95% CI: 3.69–5.01); adjusted prevalence 38.1%, (95% CI: 35.8–40.4)] compared to the highest LS7 (≥5 points) group [adjusted prevalence 16.7%, (95% CI: 15.6–17.9)]. Similarly, the odds of unilateral carotid plaques were almost two times higher in the lowest LS7 group [OR: 1.91, (95% CI: 1.65–2.22); adjusted prevalence 31.1%, (95% CI: 28.8–33.4)] compared to the highest LS7 group [adjusted prevalence 29.5%, (95% CI: 28.1–30.8)]. Supplementary Figures S3,S4 depict the association between LS7 (0–7), LS7 (0–14) and carotid plaques, respectively. Supplementary Tables S9,S10 show the ORs and adjusted prevalences of the association between LS7 (0–14) and the presence of carotid plaques.

#### Life’s Essential 8 vs. Life's Simple 7 to predict carotid plaques

**Figure 3:**
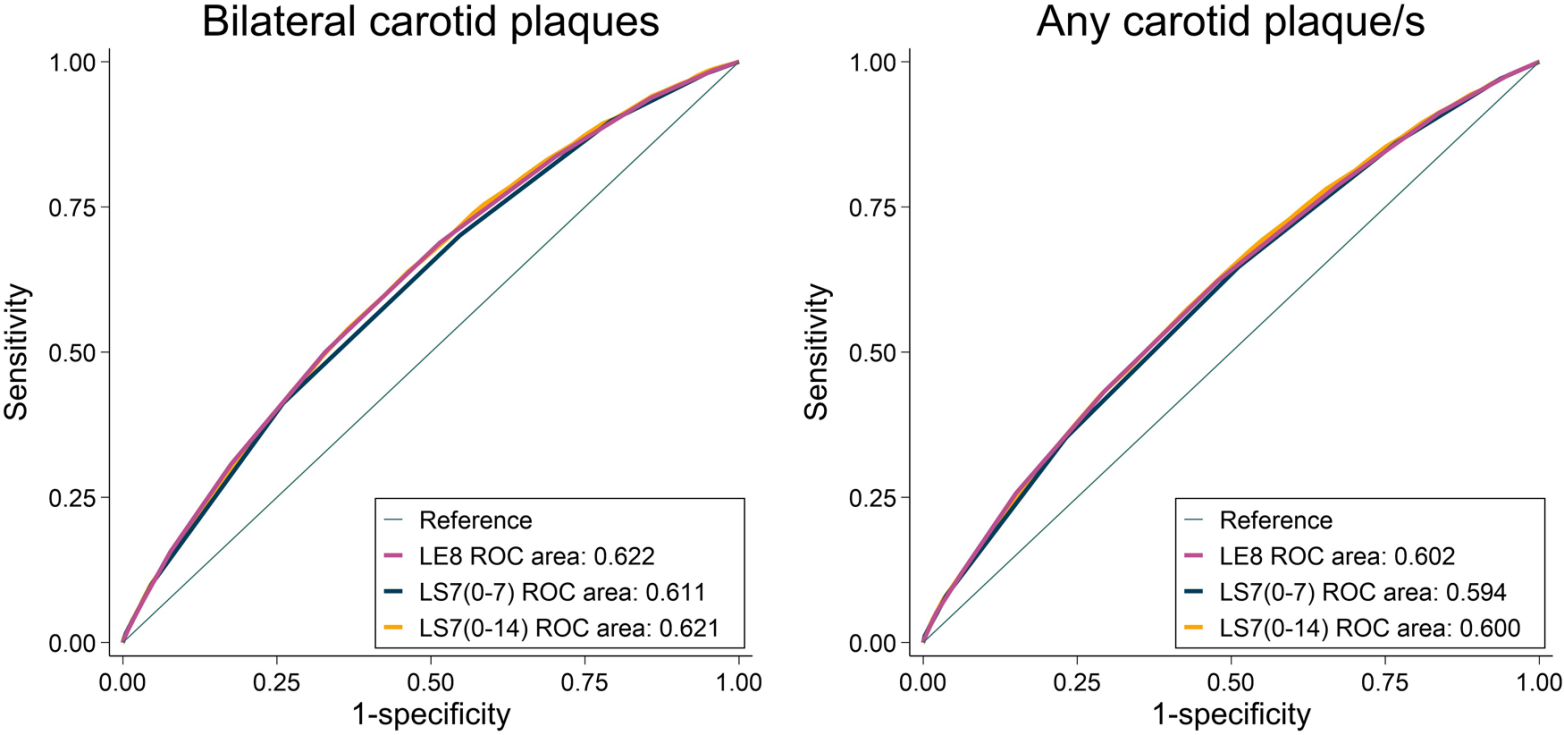
Unadjusted receiver operating characteristic (ROC) curves to predict bilateral carotid plaques and any carotid plaque/s for Life's Essential 8, Life's Simple 7 (0–7) and Life's Simple 7 (0–14).

The area under ROC curves to predict any carotid plaque was marginally higher for LE8 [0.602 (95% CI: 0.596–0.609)] compared to LS7 (0–7) [0.594 (95% CI: 0.587–0.600), *P* < 0.001]; and similar to LS7 0–14 points [0.600 (95% CI: 0.593–0.607), *P* = 0.194], respectively (Figure 3). Similarly, the area under ROC curves to predict bilateral carotid plaques was marginally higher for LE8 [0.622 (95% CI: 0.614–0.630),] compared to LS7 (0–7) [0.611 (95% CI: 0.603–0.618), *P* < 0.001]; and similar to LS7 (0–14) [0.621 (95% CI: 0.613–0.628), *P* = 0.578], respectively (Figure 3). Supplementary Table S11 depicts the reclassification and discrimination capacity of LE8, LS7 (0–7) and LS7 (0–14) beyond covariates in the Model 2 (sex, age, and site).

### Sensitivity analyses

In an extreme scenario sensitivity analysis, when all participants with missing data in either LS8 or carotid plaques were considered as having the best or the worst possible result, LS8 maintained the strong, dose-response and inverse associations with carotid plaques, though such associations were somewhat attenuated (Supplementary Table S12). In a secondary sensitivity analysis, when considering the exclusion of participants with previous stroke or cardiovascular disease, and when considering only participants with 8 components in the calculation of the LE8 score, ORs remained virtually the same (Supplementary Table S13).

## Discussion

The present study provides evidence of a strong, inverse and dose-response associations between the novel LE8 score and the presence of carotid plaques, especially bilateral ones, both in terms of relative and absolute measures. Similar results were found for the conventional LS7 score, showcasing that the LS7 (0–14) score had similar capacity to predict carotid plaques compared to LS8.

To our knowledge, this is the first study analysing the association of the novel LE8 score with carotid plaques. Our study builds on the previous literature by demonstrating the strong and inverse associations between both LS8 and LS7 scores and carotid plaques. Particularly, in our study, after adjustments, the odds for unilateral and bilateral carotid plaques were roughly two and five times higher in those with poor LE8 (<50 points) compared to those with ideal LE8 (≥80 points) scores, respectively, finding similar associations for the appurtenant LS7 score.

Although there are no previous studies to allow comparison with our LE8 results, as the construct was recently developed, our findings for LS7 are in line with those previously published in the literature. For instance, cross-sectional data from Europe and China have demonstrated inverse associations between the number of LS7 components at ideal level and carotid plaques (15, 24). Similarly, prospective studies from China have shown that a higher LS7 at baseline was associated with a lower risk of carotid plaques during follow-up (16, 25). Interestingly, CVH scores have proven to extend the association beyond subclinical atherosclerosis to cardiovascular events, in such a way that positive changes in CVH scores were associated with a reduction in the incidence of stroke (26).

In our study, in comparison to the LE8 score, the LS7 score exhibited similar capacity to predict carotid plaques, especially when LS7 was scored as 0–14 points. As such, beyond sex, site, and age, the capacity for reclassification between LE8 and LS7 (0–14) scores seems very comparable, with marginal improvements of the LE8 score to reclassify the presence of bilateral plaques and the absence of any carotid plaque/s. That is, despite the difference between LE8 and LS7 performance being statistically significant, it is not clear that this difference would have a noticeable impact on public health surveillance. This is surprising seeing as, in theory, LE8 seems more advantageous, since small changes in LS7 components or in the sleep health would go unnoticed using LS7 for follow-up in individual patients. However, in our study, sleep health was not associated with carotid plaques, which may partially explain the similar capacity of prediction between LS7 and LE8. Similarly, we did not observe any association between diet and the presence of carotid plaques. Since LE8 is a novel metric introduced in June 2022, there are no previous studies specifically analyzing its association with carotid plaques. However, a prospective cohort study investigating the association of LE8 and cardiovascular disease mortality reported that sleep health and diet had the lowest population attributable fraction among the LE8 components, which aligns with our current findings (27).

A previous study from SCAPIS showed that a Swedish version of the Systematic Coronary Risk Evaluation (SCORE) risk chart was useful to predict carotid plaques in the adult population (28). Specifically, this study proved that when doubling the SCORE, the relative probability to be in a higher carotid plaque group increased by 69%, AUC = 0.670. Such capacity of prediction was not very different from that of LE8 to predict bilateral plaques (AUC = 0.622), especially considering that LE8 does not include sex and age, two well established cardiovascular risk factors. It should be noted that LE8 (and LS7) were not conceived, at least initially, as purely cardiovascular risk predictors, but as tools to monitor cardiovascular health based on modifiable components.

In the present study, more than 55% of the SCAPIS population had carotid plaques and 25% had bilateral plaques. Due to these high prevalences, the strong relative associations (expressed as ORs) were also followed by important absolute risk metrics (expressed as adjusted prevalences). Altogether, this created a particular trade-off, with stable adjusted prevalences in unilateral plaques but dose-response prevalences in absent and bilateral plaques. This apparent discrepancy between the relative and absolute measures of association raises intriguing considerations. We have hypothesized that individuals with high and low LE8 scores may undergo opposite redistributions among the plaque groups (from bilateral to unilateral, and from unilateral to no plaques in high LE8, and vice versa in low LE8). This would result in similar prevalences of unilateral plaques among LE8 groups, but different ORs (as the prevalences of the reference category, i.e., no plaques, are different between LE8 groups). Specifically, compared to participants with ideal LE8, those with poor LE8 had adjusted prevalences that were 25.5% units lower for absent plaques, 23.4% units higher for bilateral plaques, but a neglectable 2.1% units higher for unilateral plaques. Therefore, from a clinical practice perspective, LE8 seems to be a feasible tool to monitor the absence of carotid plaques and the presence of bilateral plaques, but with poor utility to predict unilateral plaques. Nevertheless, it should be noted that bilateral plaques are more strongly associated with both stroke (29) and coronary heart disease (30) than unilateral plaques, which theoretically converts the LE8 score in a good predictor for cerebrovascular and cardiovascular events, something that the LS7 score has previously proved (10, 11). In addition, in our study, both health factors and health behaviours proved to be associated with carotid plaques. Thus, LE8, based on its simplicity and its recognizable 8 components, could serve as a personal self-monitoring tool, motivating the adherence to a better lifestyle trough easily recognized health behaviours and factors.

A main strength of this study is its size. The SCAPIS project comprises more than four times the population of relevant studies such as the MESA study (31) or the BioImaging study (32). In addition, only 4.3% of the population had missing data in the relevant variables, leaving 28,870 randomly selected participants for a complete case analysis. Finally, as recommended by the AHA, comprehensive clinical examinations including all eight LE8 components as well as imaging were performed.

However, some limitations should be recognized. First, carotid plaques were only categorized as absent/unilateral/bilateral plaques, not analysing neither the different “plaque phenotypes” nor the plaque burden. In this sense, some novel studies have proved that certain characteristics of the atherosclerotic plaques suggesting their vulnerability could improve the cardiovascular risk prediction (33). Second, despite a relatively high participation rate, and similar to most studies, areas with low socio-economic status presented lower participation rates in SCAPIS, raising some concern for selection bias (34). We speculate that this would lead to an underestimation of the association between CVH scores and subclinical atherosclerosis, as those with low socioeconomic status are likely to have a higher burden of cardiovascular disease (35). Third, our observational study had a cross-sectional design, which impedes measuring cumulative exposure in CVH scores or incident carotid plaques and drawing therefore conclusions about causality. Nonetheless, one aspect that should be considered when interpreting our findings is that our study does not concern the causal role of LE8 in carotid atherosclerosis. Instead, our study concerns and highlights the value of using LE8 as a routine monitoring tool for carotid atherosclerosis in clinical practice.

In conclusion, this large population-based study showed inverse and dose-response associations between the novel LE8 score and the presence of carotid plaques, especially bilateral ones. The conventional LS7 score showed similar capacity to predict carotid plaques, particularly when scored in a 0–14 increment scale. Both scores proved to be powerful tools to measure, monitor and promote CVH in an adult population in the clinical setting.

## Data Availability

The data analyzed in this study is subject to the following licenses/restrictions: The data underlying this article cannot be shared publicly due to legal reasons as well as the privacy of individuals that participated in the study. However, by contacting the study organization (www.scapis.org) or the corresponding author, information will be provided regarding the procedures for accessing data following Swedish legislation. Requests to access these datasets should be directed to pontus.henriksson@liu.se.
