# Supplementary material for: Life's Essential 8 and carotid artery plaques: the Swedish cardiopulmonary bioimage study
Source: Front Cardiovasc Med. 2023 Jun 22;10:1173550. doi: 10.3389/fcvm.2023.1173550 (PMC10323823; doi:10.3389/fcvm.2023.1173550)
Supplement: Supplementary file 1 [file Datasheet2.docx]

**SUPPLEMENTARY MATERIAL**

**Appendix S2**

**Life’s Essential 8 and carotid artery plaques:**

**the Swedish Cardiopulmonary Bioimage Study**

**Ángel Herraiz-Adillo^1^, Viktor H. Ahlqvist^2^, Sara Higueras-Fresnillo^1,3^, Daniel Berglind^2,4^, Patrik Wennberg^5^, Cecilia Lenander^6^, Bledar Daka^7^, Mattias Ekstedt^1,8^, Johan Sundström^9,10^, Francisco B. Ortega^11,12,13^, Carl-Johan Östgren^1,8^, Karin Rådholm^1,10^, Pontus Henriksson^1^.**

1. Department of Health, Medicine and Caring Sciences, Linköping University, Linköping, Sweden.

2. Department of Global Public Health, Karolinska Institutet, Stockholm, Sweden.

3. Department of Preventive Medicine and Public Health, Universidad Autónoma de Madrid, Madrid, Spain.

4. Centre for Epidemiology and Community Medicine, Region Stockholm, SE-10431, Stockholm, Sweden.

5. Department of Public Health and Clinical Medicine, Family Medicine, Umeå University, Umeå, Sweden.

6. Department of Clinical Sciences in Malmö, Centre for Primary Health Care Research, Lund University, Lund, Sweden.

7. School of Public Health and Community Medicine, Institute of Medicine, Sahlgrenska Academy, University of Gothenburg, Gothenburg, Sweden.

8. Centre of Medical Image Science and Visualization (CMIV), Linköping University, Linköping, Sweden.

9. Clinical Epidemiology Unit, Department of Medical Sciences, Uppsala University, Sweden.

10. The George Institute for Global Health, University of New South Wales, Sydney, Australia.

11. Department of Physical Education and Sports, Faculty of Sport Sciences, Sport and Health. University Research Institute (iMUDS), University of Granada; CIBERobn Physiopathology of Obesity and Nutrition; Granada, Spain.

12. Faculty of Sport and Health Sciences, University of Jyväskylä, Jyväskylä, Finland.

13. Department of Biosciences and Nutrition, Karolinska Institutet, Huddinge, Sweden.

**Figures**

**Figure S1.** Restricted cubic splines for the association of Life’s Essential 8 score with carotid plaques.

**Figure S2.** Life’s Simple 7 and carotid plaques.

**Figure S3.** Restricted cubic splines for the association of Life’s Simple 7 (0-7) score with carotid plaques.

**Figure S4.** Restricted cubic splines for the association of Life’s Simple 7 (0-14) score with carotid plaques.

**Tables**

**Table S1.** Clinical characteristics between the participants included and those excluded for the study.

**Table S2.** Multinomial logistic regression, associations of Life’s Essential 8 and carotid plaques.

**Table S3.** Adjusted (marginal) prevalences for the multinomial logistic regression of Life’s Essential 8 and carotid plaques.

**Table S4.** Multinomial logistic regression, associations of continuous Life’s Essential 8 (0-100 points) and carotid plaques.

**Table S5.** Multinomial logistic regression, associations of Life’s Essential 8 health behaviors and health factors and carotid plaques.

**Table S6.** Multinomial logistic regression, associations of Life’s Essential 8 components (by every 10 units increase) and carotid plaques.

**Table S7.** Multinomial logistic regression, associations of Life’s Simple 7 (0-7) and carotid plaques.

**Table S8.** Adjusted (marginal) prevalences for the multinomial logistic regression of Life’s Simple 7 (0-7) and carotid plaques.

**Table S9.** Multinomial logistic regression, associations of Life’s Simple 7 (0-14) and carotid plaques.

**Table S10.** Adjusted (marginal) prevalences for the multinomial logistic regression of Life’s Simple 7 (0-14) and carotid plaques.

**Table S11.** Net Reclassification Index and Integrated Discrimination Improvement of the Life’s Essential 8 and Life’s Simple 7 scores.

**Table S12.** Extreme scenario sensitivity analysis: multinomial logistic regression, association of Life’s Essential 8 and carotid plaques.

**Table S13.** Secondary sensitivity analyses: multinomial logistic regression, association of Life’s Essential 8 and carotid plaques.

**Figure S1.** **Restricted cubic splines for the association of Life’s Essential 8 score with carotid plaques.**

The model is based on binary logistic regression adjusted by age, sex and site: LE8 unilateral (no carotid plaque -reference- vs unilateral carotid plaques), LE8 bilateral (no carotid plaque -reference- vs bilateral carotid plaques). Reference category in LE8 score: 80 points. Y axis is in logarithmic scale.

Test for non-linearity in splines for bilateral plaques and unilateral plaque/s, p=0.458 and p=0.145, respectively.

CI: confidence interval, OR: odds ratio, LE8: Life’s Essential 8 score.

**Figure S2. Life’s Simple 7 and carotid plaques.**

Panel A illustrates the multinomial regression model to estimate odds ratios of unilateral carotid plaque and bilateral carotid plaques across Life’s Simple7 scores (adjusted for sex, age, and study site). Panel B illustrates the adjusted prevalences of unilateral carotid plaque and bilateral carotid plaques across Life’s Simple 7 scores (adjusted for sex, age, and study site).

**Figure S3.** **Restricted cubic splines for the association of Life’s Simple 7 (0-7) score with carotid plaques**.

The model is based on binary logistic regression adjusted by age, sex and site: LS7 (0-7) unilateral (no carotid plaque -reference- vs unilateral carotid plaques), LS7 (0-7) bilateral (no carotid plaque -reference- vs bilateral carotid plaques). Reference category in LS7 (0-7) score: 5 points. Y axis is in logarithmic scale.

Test for non-linearity in splines for bilateral plaques and unilateral plaque/s, p<0.001 and p=0.072, respectively.

CI: confidence interval, OR: odds ratio, LS7: Life’s Simple 7 score.

**Figure S4. Restricted cubic splines for the association of Life’s Simple 7 (0-14) score with carotid plaques**.

The model is based on binary logistic regression adjusted by age, sex and site: LS7 (0-14) unilateral (no carotid plaque -reference- vs unilateral carotid plaques), LS7 (0-14) bilateral (no carotid plaque -reference- vs bilateral carotid plaques). Reference category in LS7 (0-14) score: 12 points. Y axis is in logarithmic scale.

Test for non-linearity in splines for bilateral plaques and unilateral plaque/s, p=0.977 and p=0.547, respectively.

CI: confidence interval, OR: odds ratio, LS7: Life’s Simple 7 score.

**Table S1.** **Clinical characteristics between the participants included and those excluded for the study.**

|  | **Included participants** | | | **Excluded participants** | | |
| --- | --- | --- | --- | --- | --- | --- |
|  | **Total**  **n=28 870 (95.7%)** | **Women**  **n=14 862** | **Men**  **n=14 008** | **Total**  **n=1284 (4.3%)** | **Women**  **n=646** | **Men**  **n=638** |
| **Age and cardiovascular risk factors** | | | |  |  |  |
| Age, y | 57.5 (4.3) | 57.5 (4.3) | 57.5 (4.4) | 57.6 (4.3) | 57.7 (4.3) | 57.6 (4.3) |
| BMI, kg/m^2^ | 26.9 (4.4) | 26.5 (4.8) | 27.4 (3.9) | 27.8 (5.2) | 27.3 (5.4) | 28.3 (4.8) |
| Obesity, n (%) | 6126 (21.2) | 3036 (20.4) | 3090 (22.1) | 357 (27.9) | 165 (25.6) | 192 (30.2) |
| Total cholesterol, mg/dL | 212.2 (40.7) | 218.2 (39.5) | 205.8 (40.9) | 212.1 (41.3) | 216.8 (39.8) | 207.5 (42.3) |
| HDL cholesterol, mg/dL | 63.1 (19.3) | 71.0 (19.2) | 54.6 (15.3) | 58.2 (18.5) | 66.4 (19.0) | 51.0 (14.8) |
| LDL cholesterol, mg/dL | 133.0 (37.3) | 133.3 (37.2) | 132.6 (37.5) | 134.0 (37.1) | 133.0 (37.0) | 134.8 (37.1) |
| Hypercholesterolemia, n (%)^a^ | 3339 (11.7) | 1392 (9.5) | 1947 (14.2) | 70 (15.6) | 32 (13.7) | 38 (17.7) |
| Systolic blood pressure, mmHg | 125.8 (17.0) | 123.1 (17.8) | 128.7 (15.6) | 127.4 (17.5) | 124.2 (18.3) | 130.6 (16.2) |
| Diastolic blood pressure, mmHg | 77.5 (10.5) | 76.6 (10.8) | 78.5 (10.1) | 77.9 (10.5) | 76.3 (10.7) | 79.4 (10.0) |
| Hypertension, n (%)^a^ | 6461 (22.7) | 3079 (21.0) | 3382 (24.6) | 114 (25.4) | 49 (20.9) | 65 (30.2) |
| Fasting glucose, mg/dL | 103.2 (19.8) | 99.9 (17.1) | 106.7 (21.8) | 109.4 (31.5) | 104.7 (29.0) | 113.6 (33.0) |
| HbA1c, mmol/mol | 36.5 (6.4) | 36.2 (5.6) | 36.8 (7.1) | 38.4 (9.1) | 37.8 (8.1) | 39.0 (9.8) |
| Diabetes mellitus, n (%)^a^ | 1245 (4.4) | 459 (3.1) | 786 (5.7) | 43 (9.6) | 19 (8.1) | 24 (11.2) |
| Moderate-vigorous physical activity, min/day | 55.9 (29.8) | 54.0 (28.0) | 57.9 (31.4) | 54.3 (33.1) | 54.0 (31.0) | 54.7 (35.1) |
| LE8 diet (0-100) score | 41.1 (16.1) | 44.6 (16.2) | 37.3 (15.0) | 42.2 (16.6) | 46.5 (17.5) | 38.2 (14.7) |
| **Smoking, n (%)** |  |  |  |  |  |  |
| Current | 3665 (12.8) | 1883 (12.8) | 1782 (12.8) | 163 (36.8) | 80 (35.9) | 83 (37.7) |
| Ex-smoker | 10 393 (36.4) | 5715 (38.9) | 4678 (33.7) | 133 (30.1) | 69 (30.9) | 64 (29.1) |
| Never | 14 516 (50.8) | 7098 (48.3) | 7418 (53.5) | 147 (33.2) | 74 (33.2) | 73 (33.2) |
| **Alcohol intake, frequency** |  |  |  |  |  |  |
| Never | 2552 (8.9) | 1522 (10.3) | 1030 (7.4) | 104 (17.7) | 65 (19.3) | 39 (15.6) |
| Monthly or less | 4421 (15.5) | 2630 (17.8) | 1791 (12.9) | 121 (20.6) | 72 (21.4) | 49 (19.6) |
| 2-4 times a month | 10 951 (38.3) | 5625 (38.2) | 5326 (38.4) | 172 (29.4) | 107 (31.8) | 65 (26.0) |
| 2-3 times a week | 8606 (30.1) | 4213 (28.6) | 4393 (31.7) | 142 (24.2) | 73 (21.7) | 69 (27.6) |
| ≥4 times a week | 2070 (7.2) | 744 (5.0) | 1326 (9.6) | 4 (8.0) | 19 (5.7) | 28 (11.2) |
| **Social factors** |  |  |  |  |  |  |
| **Education level** |  |  |  |  |  |  |
| Unfinished primary school | 175 (0.6) | 86 (0.6) | 89 (0.6) | 20 (4.3) | 9 (3.7) | 11 (5.0) |
| Primary school | 2455 (8.6) | 1097 (7.4) | 1358 (9.8) | 75 (16.3) | 40 (16.5) | 35 (16.1) |
| Secondary school | 13 069 (45.6) | 6311 (42.7) | 6758 (48.6) | 202 (43.8) | 89 (36.6) | 113 (51.8) |
| University degree | 12 984 (45.3) | 7285 (49.3) | 5699 (41.0) | 164 (35.6) | 105 (43.2) | 59 (27.1) |
| **Current marital status** |  |  |  |  |  |  |
| Single | 3832 (13.4) | 2039 (13.8) | 1793 (12.9) | 110 (23.9) | 56 (23.6) | 54 (24.2) |
| Divorced | 3178 (11.1) | 2047 (13.9) | 1131 (8.2) | 72 (15.7) | 37 (15.6) | 35 (15.7) |
| Married | 21 137 (73.9) | 10 300 (69.9) | 10 837 (78.1) | 266 (57.8) | 138 (58.2) | 128 (57.4) |
| Widow | 473 (1.7) | 357 (2.4) | 116 (0.8) | 12 (2.6) | 6 (2.5) | 6 (2.7) |
| **Cardiovascular health scores** |  |  |  |  |  |  |
| **Life’s Essential 8 score** |  |  |  |  |  |  |
| <50 | 1207 (4.2) | 495 (3.3) | 712 (5.1) | 24 (16.1) | 9 (11.4) | 15 (21.4) |
| 50-59.9 | 3920 (13.6) | 1647 (11.1) | 2273 (16.2) | 21 (14.1) | 12 (15.2) | 9 (12.9) |
| 60-69.9 | 7975 (27.6) | 3517 (23.7) | 4458 (31.8) | 47 (31.5) | 19 (24.1) | 28 (40.0) |
| 70-79.9 | 8975 (31.1) | 4686 (31.5) | 4289 (30.6) | 34 (22.8) | 23 (29.1) | 11 (15.7) |
| ≥80 | 6793 (23.5) | 4517 (30.4) | 2276 (16.2) | 23 (15.4) | 16 (20.3) | 7 (10.0) |
| LE8 (0-100) score | 70.6 (11.6) | 72.6 (11.7) | 68.5 (11.1) | 65.0 (14.2) | 67.7 (14.6) | 62.1 (13.1) |
| **Life’s Simple 7 score** | | | | | | |
| LS7 (0-7) score | 3.3 (1.3) | 3.5 (1.3) | 3.0 (1.2) | 2.7 (1.4) | 2.9 (1.4) | 2.4 (1.2) |
| LS7 (0-14) score | 9.1 (2.0) | 9.5 (2.0) | 8.8 (1.9) | 8.1 (2.3) | 8.4 (2.4) | 7.7 (2.2) |
| **Carotid plaques** | | | |  |  |  |
| No plaque | 12 974 (44.9) | 7576 (51.0) | 5398 (38.5) | 435 (42.6) | 250 (49.5) | 185 (35.9) |
| Unilateral | 8583 (29.7) | 4296 (28.9) | 4287 (30.6) | 304 (29.8) | 147 (29.1) | 157 (30.4) |
| Bilateral | 7313 (25.3) | 2990 (20.1) | 4323 (30.9) | 282 (27.6) | 108 (21.4) | 174 (33.7) |
| BMI: body mass index, HDL: high density lipoprotein, LDL: low density lipoprotein, LE8: Life’s Essential 8 score, LS7: Life’s Simple 7 score.  Data refer to mean (standard deviation) and frequencies (percentage).  ^a^ Sample size in hypertension, hypercholesterolemia and diabetes mellitus, n=28 419. | | | | | | |

| **Table S2. Multinomial logistic regression, associations of Life’s Essential 8 and carotid plaques.** | | | | | | | | | |
| --- | --- | --- | --- | --- | --- | --- | --- | --- | --- |
|  | **Model 1** | | | **Model 2** | | | **Model 3** | | |
| **LE8 score** | **OR** | **(95% CI)** | **P** | **OR** | **(95% CI)** | **P** | **OR** | **(95% CI)** | **P** |
| **Bilateral plaques** | | | | | | | | | |
| **<50** | 6.41 | (5.48-7.49) | <0.001 | 4.93 | (4.19-5.79) | <0.001 | 4.59 | (3.83-5.51) | <0.001 |
| **50-59.9** | 3.99 | (3.60-4.42) | <0.001 | 3.02 | (2.71-3.35) | <0.001 | 2.84 | (2.53-3.19) | <0.001 |
| **60-69.9** | 2.79 | (2.56-3.05) | <0.001 | 2.16 | (1.98-2.37) | <0.001 | 2.10 | (1.91-2.32) | <0.001 |
| **70-79.9** | 1.81 | (1.66-1.97) | <0.001 | 1.52 | (1.39-1.67) | <0.001 | 1.52 | (1.38-1.67) | <0.001 |
| **80-100**  **(reference)** | 1.00 | - | - | 1.00 | - | - | 1.00 | - | - |
| **Unilateral plaque/s** | | | | | | | | | |
| **<50** | 2.42 | (2.07-2.85) | <0.001 | 2.14 | (1.82-2.51) | <0.001 | 2.08 | (1.74-2.50) | <0.001 |
| **50-59.9** | 1.75 | (1.59-1.92) | <0.001 | 1.52 | (1.38-1.68) | <0.001 | 1.51 | (1.36-1.68) | <0.001 |
| **60-69.9** | 1.48 | (1.37-1.60) | <0.001 | 1.31 | (1.21-1.42) | <0.001 | 1.30 | (1.20-1.42) | <0.001 |
| **70-79.9** | 1.28 | (1.19-1.37) | <0.001 | 1.18 | (1.09-1.27) | <0.001 | 1.18 | (1.09-1.28) | <0.001 |
| **80-100**  **(reference)** | 1.00 | - | - | 1.00 | - | - | 1.00 | - | - |
| CI: confidence interval, LE8 score: Life’s Essential 8 score, OR: odds ratio.  Reference category for carotid plaque: no plaque.  Model 1: unadjusted; n=28 870.  Model 2: adjusted for age, sex, and study site; n=28 870.  Model 3: adjusted for age, sex, study site, alcohol intake (frequency, and number of drinks in a typical day), educational status (achieved highest level of education), current marital status, and cardiovascular disease (self-reported myocardial infarction, coronary artery bypass grafting, percutaneous coronary intervention, stroke, or peripheral arterial disease intervention): n=25 817. | | | | | | | | | |

| **Table S3. Adjusted (marginal) prevalences for the multinomial logistic regression of Life’s Essential 8 and carotid plaques.** | | | | | | |
| --- | --- | --- | --- | --- | --- | --- |
|  | **No plaque** | | **Unilateral plaque/s** | | **Bilateral plaques** | |
| **LE8 score** | **Adjusted prevalence**  **(95% CI)** | | **Adjusted prevalence**  **(95% CI)** | | **Adjusted prevalence**  **(95% CI)** | |
| **<50** | 27.93 | (25.39-30.48) | 31.53 | (28.89-34.16) | 40.54 | (37.86-43.23) |
| **50-59.9** | 36.63 | (35.13-38.13) | 29.90 | (28.46-31.34) | 33.47 | (32.05-34.90) |
| **60-69.9** | 42.07 | (41.00-43.14) | 29.81 | (28.80-30.81) | 28.12 | (27.17-29.07) |
| **70-79.9** | 47.13 | (46.12-48.14) | 30.26 | (29.31-31.21) | 22.61 | (21.76-23.46) |
| **80-100** | 53.39 | (52.20-54.59) | 29.43 | (28.31-30.54) | 17.18 | (16.24-18.13) |
| CI: confidence interval, LE8: Life’s Essential 8 score.  The marginal prevalences are adjusted for age, sex, and study site; n= 28 870. | | | | | | |

| **Table S4. Multinomial logistic regression, associations of continuous Life’s Essential 8 (0-100 points) and carotid plaques.** | | | | |
| --- | --- | --- | --- | --- |
| **LE8 (0-100) score** | **B** | **Exp(B)** | **(95% CI)** | **P** |
| **Bilateral plaques** | | | | |
| **Model 1** | -0.046 | 0.955 | (0.952-0.957) | <0.001 |
| **Model 2** | -0.038 | 0.962 | (0.960-0.965) | <0.001 |
| **Model 3** | -0.037 | 0.964 | (0.961-0.967) | <0.001 |
| **Unilateral plaque/s** | | | | |
| **Model 1** | -0.020 | 0.980 | (0.977-0.982) | <0.001 |
| **Model 2** | -0.016 | 0.984 | (0.982-0.987) | <0.001 |
| **Model 3** | -0.015 | 0.985 | (0.982-0.987) | <0.001 |
| CI: confidence interval, LE8 score: Life’s Essential 8 score.  Reference category for carotid plaque: no plaque.  Model 1: unadjusted; n=28 870.  Model 2: adjusted for age, sex, and study site; n=28 870.  Model 3: adjusted for age, sex, study site, alcohol intake (frequency, and number of drinks in a typical day), educational status (achieved highest level of education), current marital status, and cardiovascular disease (self-reported myocardial infarction, coronary artery bypass grafting, percutaneous coronary intervention, stroke, or peripheral arterial disease intervention): n=25 817. | | | | |

| **Table S5. Multinomial logistic regression, associations of Life’s Essential 8 health behaviors and health factors and carotid plaques.** | | | | | | | | |
| --- | --- | --- | --- | --- | --- | --- | --- | --- |
|  | | **Unilateral plaque/s** | | | | **Bilateral plaques** | | |
| **LE8 health behavior score** | **n** | **OR** | **(95% CI)** | | **P** | **OR** | **(95% CI)** | **P** |
| **<50** | 1420 | 1.32 | | (1.15-1.52) | <0.001 | 2.35 | (2.06-2.70) | <0.001 |
| **50-59.9** | 2119 | 1.36 | | (1.21-1.52) | <0.001 | 2.00 | (1.78-2.24) | <0.001 |
| **60-69.9** | 4463 | 1.12 | | (1.04-1.22) | 0.005 | 1.33 | (1.22-1.45) | <0.001 |
| **70-79.9** | 7417 | 1.03 | | (0.96-1.10) | 0.398 | 1.15 | (1.07-1.24) | <0.001 |
| **80-100**  **(reference)** | 13451 | 1.00 | | - | - | 1.00 | - | - |
| **LE8 health factor score** |  |  | |  |  |  |  |  |
| **<50** | 5212 | 1.56 | | (1.43-1.70) | <0.001 | 2.95 | (2.68-3.24) | <0.001 |
| **50-59.9** | 4667 | 1.40 | | (1.29-1.53) | <0.001 | 2.33 | (2.12-2.58) | <0.001 |
| **60-69.9** | 5623 | 1.31 | | (1.20-1.42) | <0.001 | 2.11 | (1.92-2.31) | <0.001 |
| **70-79.9** | 5481 | 1.11 | | (1.02-1.21) | 0.012 | 1.62 | (1.47-1.78) | <0.001 |
| **80-100**  **(reference)** | 7887 | 1.00 | | - | - | 1.00 | - | - |
| CI: confidence interval, LE8: Life’s Essential 8 score, OR: odds ratio.  Reference category for carotid plaque: no plaque.  All models are adjusted for age, sex, and study site.  Health behaviors: nicotine exposure, physical activity, diet, and sleep health.  Health factors: blood lipids, blood pressure, fasting blood glucose and body mass index. | | | | | | | | |

| **Table S6. Multinomial logistic regression, associations of Life’s Essential 8 components (by every 10 units increase) and carotid plaques.** | | | | | | |
| --- | --- | --- | --- | --- | --- | --- |
| **LE8 components**  **(0-100)** | **Unilateral plaques** | | | **Bilateral plaques** | | |
|  | **Exp (B)** | **(95% CI)** | **P** | **Exp (B)** | **(95% CI)** | **P** |
| **Diet** | 1.015 | (0.996-1.034) | 0.126 | 1.010 | (0.989-1.032) | 0.343 |
| **Physical activity** | 0.992 | (0.958-1.028) | 0.659 | 0.942 | (0.911-0.975) | **<0.001** |
| **Nicotine exposure** | 0.963 | (0.953-0.972) | **<0.001** | 0.926 | (0.916-0.935) | **<0.001** |
| **Sleep health** | 1.004 | (0.991-1.018) | 0.549 | 0.998 | (0.984-1.013) | 0.841 |
| **BMI** | 1.019 | (1.007-1.031) | **0.001** | 1.048 | (1.035-1.062) | **<0.001** |
| **Non-HDL cholesterol** | 0.953 | (0.944-0.963) | **<0.001** | 0.908 | (0.898-0.918) | **<0.001** |
| **Fasting blood glucose/ hemoglobin A1c** | 0.991 | (0.977-1.005) | 0.202 | 0.962 | (0.947-0.977) | **<0.001** |
| **Blood pressure** | 0.951 | (0.994-0.996) | **<0.001** | 0.989 | (0.988-0.99) | **<0.001** |
| BMI: body mass index, CI: confidence interval, HDL: high density lipoprotein, LE8: Life’s Essential 8 score, OR: odds ratio.  Reference category for carotid plaque: no plaque.  All models are adjusted for age, sex, and study site. | | | | | | |

| **Table S7. Multinomial logistic regression, associations of Life’s Simple 7 (0-7) and carotid plaques.** | | | | | | | | | | | | | |
| --- | --- | --- | --- | --- | --- | --- | --- | --- | --- | --- | --- | --- | --- |
|  | **Model 1** | | | | **Model 2** | | | | | **Model 3** | | | |
| **LS7 (0-7) score** | **OR** | | **(95% CI)** | **P** | **OR** | **(95% CI)** | **P** | | | **OR** | **(95% CI)** | **P** | |
| **Bilateral plaques** | | | | | | | | | | | | | |
| **≤1** | 6.20 | (5.35-7.18) | | <0.001 | 4.30 | (3.69-5.01) | | <0.001 | 3.59 | | (3.04-4.24) | | <0.001 |
| **2** | 3.64 | (3.28-4.04) | | <0.001 | 2.59 | (2.32-2.88) | | <0.001 | 2.39 | | (2.13-2.68) | | <0.001 |
| **3** | 2.35 | (2.12-2.60) | | <0.001 | 1.84 | (1.65-2.04) | | <0.001 | 1.75 | | (1.56-1.96) | | <0.001 |
| **4** | 1.75 | (1.58-1.95) | | <0.001 | 1.52 | (1.36-1.69) | | <0.001 | 1.48 | | (1.32-1.66) | | <0.001 |
| **≥5**  **(reference)** | 1.00 | - | | - | 1.00 | - | | - | 1.00 | | - | | - |
| **Unilateral plaque/s** | | | | | | | | | | | | | |
| **≤1** | 2.28 | (1.97-2.64) | | <0.001 | 1.91 | (1.65-2.22) | | <0.001 | 1.81 | | (1.54-2.13) | | <0.001 |
| **2** | 1.68 | (1.53-1.84) | | <0.001 | 1.42 | (1.29-1.55) | | <0.001 | 1.38 | | (1.24-1.52) | | <0.001 |
| **3** | 1.45 | (1.33-1.57) | | <0.001 | 1.29 | (1.18-1.40) | | <0.001 | 1.26 | | (1.15-1.38) | | <0.001 |
| **4** | 1.21 | (1.11-1.32) | | <0.001 | 1.13 | (1.03-1.24) | | 0.008 | 1.13 | | (1.03-1.24) | | 0.012 |
| **≥5**  **(reference)** | 1.00 | - | |  | 1.00 | - | | - | 1.00 | | - | | - |
| CI: confidence interval, LS7 score: Life’s Simple 7 score, OR: odds ratio.  Reference category for carotid plaque: no plaque.  Model 1: unadjusted; n=25 881.  Model 2: adjusted for age, sex, and study site; n=25 881.  Model 3: adjusted for age, sex, study site, alcohol intake (frequency, and number of drinks in a typical day), educational status (achieved highest level of education), current marital status, and cardiovascular disease (self-reported myocardial infarction, coronary artery bypass grafting, percutaneous coronary intervention, stroke, or peripheral arterial disease intervention): n=23 220. | | | | | | | | | | | | | |

| **Table S8. Adjusted (marginal) prevalences for the multinomial logistic regression of Life’s Simple 7 (0-7) and carotid plaques.** | | | | | | |
| --- | --- | --- | --- | --- | --- | --- |
|  | **No plaque** | | **Unilateral plaque/s** | | **Bilateral plaques** | |
| **LS7 (0-7)**  **Score** | **Adjusted prevalence**  **(95% CI)** | | **Adjusted prevalence**  **(95% CI)** | | **Adjusted prevalence**  **(95% CI)** | |
| **≤1** | 30.80 | (28.49-33.10) | 31.12 | (28.82-33.42) | 38.08 | (35.77-40.39) |
| **2** | 39.63 | (38.40-40.85) | 30.04 | (28.88-31.20) | 30.34 | (29.22-31.45) |
| **3** | 44.48 | (43.37-45.59) | 30.87 | (29.82-31.92) | 24.65 | (23.70-25.60) |
| **4** | 48.22 | (46.98-49.45) | 29.54 | (28.38-30.69) | 22.25 | (21.20-23.30) |
| **≥5** | 53.82 | (52.38-55.26) | 29.46 | (28.12-30.81) | 16.71 | (15.57-17.85) |
| CI: confidence interval, LS7: Life’s Simple 7 score.  The marginal prevalences are adjusted for age, sex, and study site; n=25 881. | | | | | | |

| **Table S9. Multinomial logistic regression, associations of Life’s Simple 7 (0-14) and carotid plaques.** | | | | | | | | | | | | |
| --- | --- | --- | --- | --- | --- | --- | --- | --- | --- | --- | --- | --- |
|  | **Model 1** | | | | **Model 2** | | | | **Model 3** | | | |
| **LS7 score (0-14)** | **OR** | | **(95% CI)** | **P** | **OR** | **(95% CI)** | **P** | | **OR** | **(95% CI)** | **P** | |
| **Bilateral plaques** | | | | | | | | | | | | |
| **≤5** | 8.00 | (6.61-9.68) | | <0.001 | 5.50 | (4.52-6.69) | <0.001 | 4.64 | | (3.75-5.74) | | <0.001 |
| **6-7** | 4.96 | (4.36-5.64) | | <0.001 | 3.51 | (3.07-4.01) | <0.001 | 3.26 | | (2.82-3.76) | | <0.001 |
| **8-9** | 3.06 | (2.72-3.45) | | <0.001 | 2.29 | (2.02-2.59) | <0.001 | 2.19 | | (1.93-2.50) | | <0.001 |
| **10-11** | 1.84 | (1.63-2.08) | | <0.001 | 1.53 | (1.35-1.74) | <0.001 | 1.50 | | (1.32-1.71) | | <0.001 |
| **≥12**  **(reference)** | 1.00 | - | | - | 1.00 | - | - | 1.00 | | - | | - |
| **Unilateral plaque/s** | | | | | | | | | | | | |
| **≤5** | 2.78 | (2.31-3.35) | | <0.001 | 2.32 | (1.92-2.80) | <0.001 | 2.17 | | (1.77-2.66) | | <0.001 |
| **6-7** | 1.98 | (1.78-2.21) | | <0.001 | 1.68 | (1.50-1.87) | <0.001 | 1.64 | | (1.45-1.85) | | <0.001 |
| **8-9** | 1.59 | (1.44-1.75) | | <0.001 | 1.38 | (1.25-1.52) | <0.001 | 1.37 | | (1.23-1.52) | | <0.001 |
| **10-11** | 1.32 | (1.20-1.45) | | <0.001 | 1.21 | (1.10-1.33) | <0.001 | 1.21 | | (1.09-1.34) | | <0.001 |
| **≥12**  **(reference)** | 1.00 | - | | - | 1.00 | - | - | 1.00 | | - | | - |
| CI: confidence interval, LS7 score: Life’s Simple 7 score, OR: odds ratio.  Reference category for carotid plaque: no plaque.  Model 1: unadjusted; n=25 881.  Model 2: adjusted for age, sex, and study site; n=25 881.  Model 3: adjusted for age, sex, study site, alcohol intake (frequency, and number of drinks in a typical day), educational status (achieved highest level of education), current marital status, and cardiovascular disease (self-reported myocardial infarction, coronary artery bypass grafting, percutaneous coronary intervention, stroke, or peripheral arterial disease intervention): n=23 230. | | | | | | | | | | | | |

| **Table S10. Adjusted (marginal) prevalences for the multinomial logistic regression of Life’s Simple 7 (0-14) and carotid plaques.** | | | | | | |
| --- | --- | --- | --- | --- | --- | --- |
|  | **No plaque** | | **Unilateral plaque/s** | | **Bilateral plaques** | |
| **LS7 (0-14)**  **Score** | Adjusted prevalence  (95% CI) | | Adjusted prevalence  (95% CI) | | Adjusted prevalence  (95% CI) | |
| **≤5** | 28.33 | (25.45-31.21) | 32.29 | (29.33-35.26) | 39.38 | (36.41-42.35) |
| **6-7** | 36.47 | (35.05-37.88) | 30.38 | (29.01-31.74) | 33.16 | (31.82-34.50) |
| **8-9** | 43.44 | (42.44-44.45) | 30.16 | (29.21-31.11) | 26.40 | (25.52-27.28) |
| **10-11** | 49.27 | (48.21-50.33) | 30.24 | (29.24-31.23) | 20.49 | (19.62-21.36) |
| **≥12** | 55.89 | (54.13-57.64) | 28.65 | (27.02-30.28) | 15.46 | (14.10-16.82) |
| CI: confidence interval, LS7: Life’s Simple 7 score.  The marginal prevalences are adjusted for age, sex, and study site; n=25 881. | | | | | | |

| **Table S11. Net Reclassification Index and Integrated Discrimination Improvement of the Life’s Essential 8 and Life’s Simple 7 scores.** | | | | | | | | | |
| --- | --- | --- | --- | --- | --- | --- | --- | --- | --- |
|  | **NRI** | **Standard Error** | **NRI_**  **event** | **NRI_**  **No-event** | | **p** | **IDI** | **Standard Error** | **p** |
| **Bilateral plaque** | | | | | | | | | |
| Model 2 | NA | NA | NA | NA | NA | | NA | NA | NA |
| Model 2 + LE8 | 0.0446 | 0.0031 | 0.0619 | -0.0173 | <0.001 | | 0.0225 | 0.0010 | <0.001 |
| Model 2 + LS7 (0-7) | 0.0253 | 0.0025 | 0.0361 | -0.0108 | <0.001 | | 0.0161 | 0.0009 | <0.001 |
| Model 2 + LE7 (0-14) | 0.0395 | 0.0031 | 0.0563 | -0.0168 | <0.001 | | 0.0209 | 0.0010 | <0.001 |
| **Any plaque/s** | | | | | | | | | |
| Model 2 | NA | NA | NA | NA | NA | | NA | NA | NA |
| Model 2 + LE8 | 0.0386 | 0.0048 | -0.0077 | 0.0462 | <0.001 | | 0.0187 | 0.0008 | <0.001 |
| Model 2 + LS7 (0-7) | 0.0266 | 0.0050 | -0.0019 | 0.0285 | <0.001 | | 0.0142 | 0.0008 | <0.001 |
| Model 2 + LE7 (0-14) | 0.0348 | 0.0051 | -0.0063 | 0.0412 | <0.001 | | 0.0175 | 0.0008 | <0.001 |
| IDI, Integrated Discrimination Improvement; LE8, Life's Essential 8; LS7, Life's Simple 7; NA, not applicable; NRI, Net Reclassification Index.  For NRI, the threshold level for the risk categories was set at 50%.  Model 2: sex, age, and site.  Model 2 + LE8 tests the NRI of the LE8 score when added to a model that includes sex, age, and site.  Model 2 + LS7 (0-7) tests the NRI of the LS7 (0-7) score when added to a model that includes sex, age, and site.  Model 2 + LS7 (0-14) tests the NRI of the LS7 (0-14) score when added to a model that includes sex, age, and site. | | | | | | | | | |

| **Table S12. Extreme scenario sensitivity analysis: multinomial logistic regression, association of Life’s Essential 8 and carotid plaques.** | | | | | | | | | | | | | | | | | | | |
| --- | --- | --- | --- | --- | --- | --- | --- | --- | --- | --- | --- | --- | --- | --- | --- | --- | --- | --- | --- |
|  | | **Best LE8, carotid plaque**  **n=29 891** | | | **Worst LE8, carotid plaque**  **n=29 891** | | | **LE8, best carotid plaque**  **n=29 019** | | | **LE8, worst carotid plaque**  **n=29 019** | | | **Best LE8, best carotid plaque**  **n=30 154** | | | **Worst LE8, worst carotid plaque**  **n=30 154** | | |
| **Unilateral**  **plaques** | **LE8**  **score** | OR | (95% CI) | P | OR | (95% CI) | P | OR | (95% CI) | P | OR | (95% CI) | P | OR | (95% CI) | P | OR | (95% CI) | P |
|  | **<50** | 2.08 | (1.77-2.44) | <0.001 | 1.62 | (1.44-1.82) | <0.001 | 1.99 | (1.69-2.33) | <0.001 | 2.13 | 1.82 | <0.001 | 1.99 | (1.70-2.33) | <0.001 | 1.62 | (1.44-1.83) | <0.001 |
|  | **50-59.9** | 1.48 | (1.35-1.63) | <0.001 | 1.52 | (1.38-1.68) | <0.001 | 1.51 | (1.37-1.66) | <0.001 | 1.52 | 1.38 | <0.001 | 1.51 | (1.37-1.66) | <0.001 | 1.52 | (1.38-1.68) | <0.001 |
|  | **60-69.9** | 1.28 | (1.18-1.37) | <0.001 | 1.31 | (1.21-1.41) | <0.001 | 1.30 | (1.20-1.40) | <0.001 | 1.31 | 1.21 | <0.001 | 1.30 | (1.21-1.40) | <0.001 | 1.31 | (1.21-1.41) | <0.001 |
|  | **70-79.9** | 1.15 | (1.07-1.23) | <0.001 | 1.18 | (1.09-1.27) | <0.001 | 1.17 | (1.09-1.26) | <0.001 | 1.18 | 1.09 | <0.001 | 1.18 | (1.1-1.26) | <0.001 | 1.18 | (1.09-1.27) | <0.001 |
|  | **80-100**  **(Ref.)** | 1.00 | - | - | 1.00 | - | - | 1.00 | - | - | 1.00 | - | - | 1.00 | - | - | 1.00 | - | - |
| **Bilateral plaques** | **LE8**  **score** |  |  |  |  |  |  |  |  |  |  |  |  |  |  |  |  |  |  |
|  | **<50** | 4.40 | (3.75-5.15) | <0.001 | 3.23 | (2.85-3.66) | <0.001 | 4.57 | (3.90-5.35) | <0.001 | 5.06 | (4.32-5.94) | <0.001 | 4.21 | (3.60-4.92) | <0.001 | 3.74 | (3.31-4.23) | <0.001 |
|  | **50-59.9** | 2.69 | (2.43-2.97) | <0.001 | 3.01 | (2.71-3.34) | <0.001 | 2.98 | (2.68-3.31) | <0.001 | 3.01 | (2.71-3.35) | <0.001 | 2.75 | (2.48-3.04) | <0.001 | 3.01 | (2.71-3.35) | <0.001 |
|  | **60-69.9** | 1.93 | (1.77-2.1) | <0.001 | 2.16 | (1.97-2.36) | <0.001 | 2.14 | (1.95-2.34) | <0.001 | 2.17 | (1.98-2.37) | <0.001 | 1.97 | (1.81-2.14) | <0.001 | 2.17 | (1.98-2.37) | <0.001 |
|  | **70-79.9** | 1.36 | (1.25-1.48) | <0.001 | 1.52 | (1.39-1.66) | <0.001 | 1.52 | (1.39-1.66) | <0.001 | 1.52 | (1.39-1.66) | <0.001 | 1.40 | (1.29-1.52) | <0.001 | 1.52 | (1.39-1.66) | <0.001 |
|  | **80-100**  **(Ref.)** | 1.00 | - | - | 1.00 | - | - | 1.00 | - | - | 1.00 | - | - | 1.00 | - | - | 1.00 | - | - |
| CI: confidence interval, LE8: Life’s Essential 8 score, OR: odds ratio, Ref.: reference.  Reference group: no carotid plaque.  Models:  Best LE8, carotid plaque: missing values in LE8 score are considered as 80-100 score (ideal cardiovascular health).  Worst LE8, carotid plaque: missing values in LE8 score are considered <50 score 0 (poor cardiovascular health).  LE8, best carotid plaque: missing values in carotid plaque are considered as “no carotid plaque”.  LE8, worst carotid plaque: missing values in carotid plaque are considered as “bilateral carotid plaque”.  Best LE8, best carotid plaque: missing values in LE8 score are considered as 80-100 score (ideal cardiovascular health), missing values in carotid plaque are considered as “no carotid plaque”.  Worst LE8, worst carotid plaque: missing values in LE8 score are considered as <50 score (poor cardiovascular health), missing values in carotid plaque are considered as “bilateral carotid plaque”.  All models are adjusted for age, sex, and study site. | | | | | | | | | | | | | | | | | | | |

| **Table S13. Secondary sensitivity analyses: multinomial logistic regression, association of Life’s Essential 8 and carotid plaques.** | | | | | | | | | | | | | | |
| --- | --- | --- | --- | --- | --- | --- | --- | --- | --- | --- | --- | --- | --- | --- |
|  | | **Main analysis**  **n=28 870** | | | | **Exclusion of stroke**  **n=28 470** | | | **Exclusion of CVD**  **n=27 847** | | | **Considering 8 components**  **in LE8 (instead of 7 or 8).**  **n=25 637** | | |
|  | | OR (95% CI) | | | P | OR (95% CI) | | P | OR (95% CI) | | P | OR (95% CI) | | P |
| **Unilateral**  **plaques** | **LE8 score** |  |  |  | |  |  |  |  |  |  |  |  |  |
|  | **<50** | 2.14 | (1.82-2.51) | <0.001 | | 2.17 | (1.84-2.55) | <0.001 | 2.16 | (1.83-2.55) | <0.001 | 2.11 | (1.76-2.54) | <0.001 |
|  | **50-59.9** | 1.52 | (1.38-1.68) | <0.001 | | 1.53 | (1.38-1.68) | <0.001 | 1.52 | (1.37-1.67) | <0.001 | 1.56 | (1.41-1.73) | <0.001 |
|  | **60-69.9** | 1.31 | (1.21-1.42) | <0.001 | | 1.32 | (1.22-1.42) | <0.001 | 1.32 | (1.22-1.43) | <0.001 | 1.34 | (1.24-1.46) | <0.001 |
|  | **70-79.9** | 1.18 | (1.09-1.27) | <0.001 | | 1.18 | (1.10-1.27) | <0.001 | 1.17 | (1.09-1.27) | <0.001 | 1.18 | (1.09-1.27) | <0.001 |
|  | **80-100**  **(Ref.)** | 1.00 | - | - | | 1.00 | - | - | 1.00 | - | - | 1.00 | - | - |
| **Bilateral plaques** | **LE8 score** |  |  |  | |  |  |  |  |  |  |  |  |  |
|  | **<50** | 4.93 | (4.19-5.79) | <0.001 | | 4.92 | (4.18-5.79) | <0.001 | 4.86 | (4.12-5.74) | <0.001 | 4.84 | (4.03-5.80) | <0.001 |
|  | **50-59.9** | 3.02 | (2.71-3.35) | <0.001 | | 3.02 | (2.71-3.36) | <0.001 | 2.98 | (2.68-3.32) | <0.001 | 2.96 | (2.64-3.31) | <0.001 |
|  | **60-69.9** | 2.16 | (1.98-2.37) | <0.001 | | 2.18 | (1.99-2.39) | <0.001 | 2.17 | (1.98-2.38) | <0.001 | 2.12 | (1.92-2.33) | <0.001 |
|  | **70-79.9** | 1.52 | (1.39-1.67) | <0.001 | | 1.53 | (1.40-1.68) | <0.001 | 1.52 | (1.39-1.67) | <0.001 | 1.49 | (1.35-1.64) | <0.001 |
|  | **80-100**  **(Ref.)** | 1.00 | - | - | | 1.00 | - | - | 1.00 | - | - | 1.00 | - | - |
| CI: confidence interval, CVD: cardiovascular disease, LE8: Life’s Essential 8, OR: odds ratio, Ref.: reference.  Reference group: no carotid plaque.  CVD group refers to participant with self-reported myocardial infarction, coronary artery bypass grafting, percutaneous coronary intervention, stroke, or peripheral arterial disease intervention.  All models refer to adjusted models for age, sex, and study site. | | | | | | | | | | | | | | |
