# Supplementary material for: Life's Essential 8 and carotid artery plaques: the Swedish cardiopulmonary bioimage study
Source: Front Cardiovasc Med. 2023 Jun 22;10:1173550. doi: 10.3389/fcvm.2023.1173550 (PMC10323823; doi:10.3389/fcvm.2023.1173550)
Supplement: Supplementary file 2 [file Datasheet1.pdf]

SUPPLEMENTARY MATERIAL

Appendix S1

Life's Essential 8 and carotid artery plaques: the Swedish Cardiopulmonary Bioimage Study

Ángel Herraiz-Adillo[1], Viktor H. Ahlqvist[2], Sara Higueras-Fresnillo[1,3], Daniel Berglind[2,4], Patrik Wennberg[5], Cecilia Lenander[6], Bledar Daka[7], Mattias Ekstedt[1,8], Johan Sundström[9,10], Francisco B. Ortega[11,12,13], Carl-Johan Östgren[1,8], Karin Rådholm[1,10], Pontus Henriksson[1].

1. Department of Health, Medicine and Caring Sciences, Linköping University, Linköping, Sweden.

2. Department of Global Public Health, Karolinska Institutet, Stockholm, Sweden.

3. Department of Preventive Medicine and Public Health, Universidad Autónoma de Madrid, Madrid, Spain.

4. Centre for Epidemiology and Community Medicine, Region Stockholm, SE-10431, Stockholm, Sweden.

5. Department of Public Health and Clinical Medicine, Family Medicine, Umeå University, Umeå, Sweden.

6. Department of Clinical Sciences in Malmö, Centre for Primary Health Care Research, Lund University, Lund, Sweden.

7. School of Public Health and Community Medicine, Institute of Medicine, Sahlgrenska Academy, University of Gothenburg, Gothenburg, Sweden.

8. Centre of Medical Image Science and Visualization (CMIV), Linköping University, Linköping, Sweden.

9. Clinical Epidemiology Unit, Department of Medical Sciences, Uppsala University, Sweden.

10. The George Institute for Global Health, University of New South Wales, Sydney, Australia.

11. Department of Physical Education and Sports, Faculty of Sport Sciences, Sport and Health. University Research Institute (iMUDS), University of Granada; CIBERobn Physiopathology of Obesity and Nutrition; Granada, Spain.

12. Faculty of Sport and Health Sciences, University of Jyväskylä, Jyväskylä, Finland.

13. Department of Biosciences and Nutrition, Karolinska Institutet, Huddinge, Sweden.

Measurement and calculation of health factors and health behaviors in Life´s Essential 8

Health factors

In brief, Life´s Essential 8 (LE8) factors were measured using standardized clinical and laboratory procedures. For blood glucose and lipids, baseline biochemistry levels were evaluated through the analysis of freshly obtained blood samples collected after an overnight fast, utilizing the Architect Abbott® and Cobas Rocher® analyzers at laboratories located within the specific university hospitals.

Data about the prescription of antihypertensive (Anatomical Therapeutic Codes [ATC] codes C02, C03, C07, C08, and C09), lipid lowering (ATC code C10) and antidiabetic (ATC code A10) medications during the year prior to measurements were collected from the Swedish Prescribed Drug Register. This register is a database that records all prescription drugs dispensed to individuals in Sweden since July 2005, with an estimated national coverage rate of 100%.

Body mass index

Body mass index (BMI) was measured using standardized methods as Kg/m^2.

BMI (kg/m^2)	Score
<25.0	100
25.0-29.9	70
30.0-34.9	30
35.0-39.9	15
≥40.0	0

Blood lipids

Non-high density lipoprotein (non-HDL) cholesterol was calculated as total cholesterol – HDL cholesterol.

Non-HDL cholesterol (mg/dl)	Score
<130.0	100
130.0-159.9	60
160.0-189.9	40
190.0-219.9	20
≥220.0	0
If drug-treated level, 20 points were subtracted.	

Blood glucose

Venous plasma glucose was considered by default. When venous plasma was not available (2703 participants), data from capillary whole-blood samples were used, after converting to plasma capillary glucose by multiplying whole-blood capillary samples by 1.11 according to the International Federation of Clinical Chemistry and Laboratory Medicine (IFCC). Capillary samples were analysed with HemoCue® Glucose 201RT Systems glucometers based on the glucose dehydrogenase-method[1].

Fasting blood glucose (FBG) (mg/dl), hemoglobin A1c (HbA1c) (%)	Score
No history of diabetes and FBG <100.0 (or HbA1c <5.70)	100
No diabetes and FBG 100.0-125.9 (or HbA1c 5.70-6.49) (prediabetes)	60
Diabetes with HbA1c <7.0	40
Diabetes with HbA1c 7.0-7.9	30
Diabetes with HbA1c 8.0-8.9	20
Diabetes with Hb A1c 9.0-9.9	10
Diabetes with HbA1c ≥10.0	0
Diabetes was considered as: 1) taking antidiabetic medication, or HbA1c ≥6.50%, or FBG ≥126.0 mg/dl.	

Blood pressure

Systolic and diastolic brachial arterial pressures (SBP and DBP) were obtained by automatic measurement in both arms with the oscillometer Omron M10-IT® (Omron Health care Co, Kyoto, Japan)[2]. After 5 minutes rest in the supine position, the cuff-pressure was located at heart level and SBP and DBP were registered. The measurement was repeated with at least one minute between measurements. If the two results differed >10 mmHg in the same arm, either for SBP or DBP, the measurement was repeated until two subsequent results were within ±10 mmHg, with a maximum of four attempts. If the difference was still >10 mmHg, the last two measurements were considered. The measurements were performed before administration of beta-stimulants (for spirometry) or beta-blockers (for Computed Coronary Tomography Angiography [CCTA] evaluation) or on a different day. After measuring twice in each arm, the average pressure of the arm with the highest blood pressure was considered for the analysis.

For blood pressure computation, the worst scenario is considered, e.g., a patient with SBP=145.0 mmHg and DBP=85.0 mmHg scores 25 points.

SBP and DBP (mmHg)	Score
<120.0/<80.0	100
120.0–129.9/<80.0	75
130.0–139.9 or 80.0–89.9	50
140.0–159.9 or 90.0–99.9	25
≥160.0 or ≥100.0	0
If drug-treated level 20 points were subtracted.	

<u>Health behaviors</u>

Diet

The evaluation of dietary habits was conducted using the web-based MiniMeal-Q questionnaire[3,4].

The scoring of dietary habits was aligned with the Mediterranean Eating Pattern for Americans (MEPA)[5].

Diet Component	Scoring criteria
Olive oil	Consumers of olive oil: 1 point
Green leafy vegetables	Consuming ≥7 times per week: 1 point
Other vegetables	Consuming ≥2 times per day: 1 point
Berries	Consuming ≥2 times per week: 1 point
Other fruit	Consuming ≥2 times per day: 1 point
Meat	Consuming ≤3 times per week red meat, hamburger, bacon, or sausage: 1 point
Fish	Consuming ≥1 times per week: 1 point
Chicken	Consuming ≤5 times per week: 1 point
Cheese	Consuming ≤4 servings per week: 1 point
Butter/cream	Consuming <2 of these 3 food items: cooking butter, spreading butter, or rapeseed oil-butter: 1 point
Beans	Consuming ≥3 times per week: 1 point
Whole grains	Consuming ≥3 servings per day: 1 point
Sweets and pastries	Consuming ≤4 servings per week: 1 point
Nuts	Consuming ≥4 times per week: 1 point
Fast food	Consuming ≤4 meals at restaurants per week: 1 point
Alcohol	Consuming >0 or ≤2 servings of alcohol per day for men and >0 or ≤1 servings of alcohol per day for women: 1 point
**Diet total score**	Diet total score is computed when 12, 13, 14, 15 or 16 subcomponents are present. Diet total score is corrected as follows:

Corrected diet total score = (Diet total score x 16) / (16-Total subcomponents missed in diet total score).

Corrected diet total score	Points
15.0-16.0	100
12.0-14.9	80
8.0-11.9	50
4.0-7.9	25
0-3.9	0

Physical activity

Physical activity was measured through tri-axial accelerometry using the Actigraph GT3X+ (3% of participants), wGT3X+ (15% of participants) and wGT3X-BT (82% of participants) devices with low-frequency extension filter (ActiGraph LCC, Pensacola, FL, USA). Participants were instructed to wear the accelerometer on their right hip for a period of seven consecutive days during "all wake time", excluding water-based activities[6].

A categorical classification system was applied to assess physical activity levels, with the following criteria: low-intensity physical activity (200-2689 counts per minute), moderate-intensity physical activity (2690-6166 counts per minute), and vigorous-intensity physical activity (≥6167 counts per minute)[7].

For the purpose of calculation, each minute of moderate activity was recorded as one minute and each minute of vigorous activity was recorded as two minutes.

Moderate (or greater) intensity activity per week (minutes)	Score
≥150	100
120-149	90
90-119	80
60-89	60
30-59	40
1-29	20
0	0

Nicotine exposure

Smoking habit was evaluated using a questionnaire recruiting information about 1) self-reported current and former smoking status (regular and occasional smokers are both classified as current smokers), 2) age at the initiation of smoking, 3) age of cessation of smoking, 4) pack of cigarettes per year, calculated as (average daily cigarette consumption during years of smoking x number of years of smoking)/cigarettes per package, and 5) cohabitants smoking at home.

Combustible tobacco use or inhaled nicotine-delivery system (NDS) use; or second-hand smoke exposure	Score
Never smoker	100
Former smoker, quit ≥5 years	75
Former smoker, quit 1-<5 years	50
Former smoker, quit <1 years, or currently using inhaled NDS	25
Current smoker	0
Assumption: smokers who did not answer the questions on consumption of any type of tobacco were assumed to consume the median value for each category (6 for occasional smokers, 15 for all other current smokers, and 10 for ex-smokers).<br>In those participants with cohabitant smoking (or who smoked) less than 10 years, 10-20 years and >20 years, 10, 15 and 20 points were subtracted to the nicotine exposure score, respectively.	

Sleep health

Sleep patterns were evaluated through self-administered questionnaire which included the following aspects: hours of sleep per night, breathing problems during sleep (self-reported or others-reported), sleep apnea (doctor-diagnosed or self-reported), and sleep apnea treatment (self-reported) were reported from self-administered questionnaire.

Sleep per night (average hours)	Score
7-<9	100
9-<10	90
6-<7	70
5-<6 or ≥10	40
4-<5	20
<4	0
In those participants with sleep apnea or breathing problems during sleep and without receiving treatment, 20 points were subtracted for the sleep health score.	

Total Life´s Essential 8 score calculation

The determination of scores in LE8 aligns with the methodology published by the American Heart Association (AHA)[8]. In accordance with the AHA, the 8 components of LE8 are scored on a scale ranging from 0 (lowest possible level) to 100 (highest possible level).

In this study, the calculation of LE8 was performed when either 8 or 7 components were available. The LE8 score was calculated as the unweighted mean of the present components, yielding a total score in the range of 0-100 that was adjusted for the number of missing components as follows:

- For participants with 8 available components, LE8 = sum of all 8 components / 8.
- For participants with 7 available components, LE8 = sum of all 7 components / 7.

Furthermore, two separate scores were determined for the LE8 factors and the LE8 behaviors. In both cases, the scores were calculated when 3 or 4 components were available and corrected for the missing components using the same methodology as previously described.

Measurement and calculation of health factors and health behaviors in Life´s Simple 7

Though the calculation of scores is different between LE8 and Life´s Simple 7 (LS7) scores, the measurements used in LS7 score were similar to those used in LE8 score (apart from sleep health that is not included in LS7).

Diet

The diet component was consistent with a Dietary Approaches to Stop Hypertension (DASH) eating plan, as the AHA recommended[9].

Diet Subcomponents	Scoring criteria in DASH	Assumptions
Fruit and vegetables	≥4.5 cups per day	Cups per day=times per day
Fish	≥two 3.5 oz servings per week	Servings per day=times per day
Fiber-rich whole grains	≥1.1 g of fiber per 10 g of carbohydrate: ≥three 1 oz-equivalent servings per day	Goal: ≥85 g of total whole grain per day
Sodium	<1500 mg per day	
Sugar-sweetened beverages	≤450 kcal (36 oz) per week	Goal: ≤3 times per week

Diet total score: only those participants with data in all 5 sub-components are considered. All 5 subcomponents were scaled to 2000 Kcal/day.

DASH: Dietary Approaches to Stop Hypertension, g: gram, mg: milligram, oz: ounce.

Life´s Simple 7 score: definition of Poor, Intermediate and Ideal Cardiovascular Health (modified from the American Heart Association[9])

METRICS	DEFINITIONS
**Health Factors**	
Total cholesterol	*Ideal*: <5.2 mmol/L (US: <200 mg/dL) untreated<br>*Intermediate*: 5.2-6.1 mmol/L (US: 200-239 mg/dL) or treated to goal<br>*Poor*: ≥6.2 mmol/L (US: ≥240 mg/dL)
Blood pressure	*Ideal*: SBP <120 mmHg and DBP <80 mmHg untreated<br>*Intermediate*: SBP 120-139 mmHg or DBP 80-89 mmHg or treated to goal<br>*Poor*: DBP ≥140 mmHg or DBP ≥90 mmHg
Fasting blood glucose	*Ideal*: <5.6 mmol/L (<100 mg/dL) untreated<br>*Intermediate*: 5.6-6.9 mmol/L (100-125 mg/dL) or treated to goal<br>*Poor*: ≥7.0 mmol/L (US: ≥126 mg/dL)
**Health Behaviors**	
Smoking status	*Ideal*: Never smoker or quit >12 months<br>*Intermediate*: Former smoker ≤12 months<br>*Poor*: Current smoker
Body Mass Index	*Ideal*: <25.0 kg/m^2<br>*Intermediate*: 25.0-29.9 kg/m^2<br>*Poor*: ≥30.0 kg/m^2
Physical activity	*Ideal*: ≥150 min/week of moderate-intensity or ≥75 min/week of vigorous-intensity or ≥150 min/week of moderate-vigorous intensity combination<br>*Intermediate*: 1-149 min/week of moderate-intensity or 1-74 min/week of vigorous-intensity or 1-149 min/week of moderate-vigorous intensity combination<br>*Poor*: None
Diet	*Ideal*: 4-5 components<br>*Intermediate*: 2-3 components<br>*Poor*: 0-1 component

SBP: systolic blood pressure; DBP: diastolic blood pressure, US: United States standard.

Total Life´s Simple 7 score calculation

The calculation of scores in LS7 are consistent with those published by the AHA[9].

In the total score, only those participants with data in all 7 components are considered.

Two scores of LS7 were created:

- LS7 (0-7) score, we calculated the number of LS7 components at ideal level, thus creating a score ranging from 0 (the lowest cardiovascular health) to 7 (the highest cardiovascular health) points.
- LS7 (0-14) score, we calculated a combined score of the 7 components which were rated as 0 (poor cardiovascular health), 1 (intermediate cardiovascular health), or 2 (ideal cardiovascular health), leaving a total score from 0 (the lowest CVH) to 14 (the highest CVH) points.

References

1. D'Orazio P, Burnett RW, Fogh-Andersen N, Jacobs E, Kuwa K, Külpmann WR, et al. Approved IFCC recommendation on reporting results for blood glucose: International Federation of Clinical Chemistry and Laboratory Medicine Scientific Division, Working Group on Selective Electrodes and Point-of-Care Testing (IFCC-SD-WG-SEPOCT). Clin Chem Lab Med. 2006;44(12):1486-90.

2. Williams B, Mancia G, Spiering W, Agabiti Rosei E, Azizi M, Burnier M, et al. 2018 ESC/ESH Guidelines for the management of arterial hypertension: The Task Force for the management of arterial hypertension of the European Society of Cardiology and the European Society of Hypertension: The Task Force for the management of arterial hypertension of the European Society of Cardiology and the European Society of Hypertension. J Hypertens. 2018;36(10):1953-2041.

3. Nybacka S, Bertéus Forslund H, Wirfält E, Larsson I, Ericson U, Warensjö Lemming E, et al. Comparison of a web-based food record tool and a food-frequency questionnaire and objective validation using the doubly labelled water technique in a Swedish middle-aged population. J Nutr Sci. 2016;5:e39.

4. Christensen SE, Möller E, Bonn SE, Ploner A, Wright A, Sjölander A, et al. Two new meal- and web-based interactive food frequency questionnaires: validation of energy and macronutrient intake. J Med Internet Res. 2013;15(6):e109.

5. Cerwinske LA, Rasmussen HE, Lipson S, Volgman AS, Tangney CC. Evaluation of a dietary screener: the Mediterranean Eating Pattern for Americans tool. *J Hum Nutr Diet*. 10 2017;30(5):596-603. doi:10.1111/jhn.12451.

6. Ekblom-Bak E, Börjesson M, Bergman F, Bergström G, Dahlin-Almevall A, Drake I, et al. Accelerometer derived physical activity patterns in 27.890 middle-aged adults: The SCAPIS cohort study. Scand J Med Sci Sports. 2022;32(5):866-80.

7. Sasaki JE, John D, Freedson PS. Validation and comparison of ActiGraph activity monitors. J Sci Med Sport. 2011;14(5):411-6.

8. Lloyd-Jones DM, Allen NB, Anderson CAM, et al. Life's Essential 8: Updating and Enhancing the American Heart Association's Construct of Cardiovascular Health: A Presidential Advisory From the American Heart Association. *Circulation*. Aug 02 2022;146(5):e18-e43. doi:10.1161/CIR.0000000000001078.

9. Lloyd-Jones DM, Hong Y, Labarthe D, et al. Defining and setting national goals for cardiovascular health promotion and disease reduction: the American Heart Association's strategic Impact Goal through 2020 and beyond. *Circulation*. Feb 02 2010;121(4):586-613. doi:10.1161/CIRCULATIONAHA.109.192703.
